# IPMK Mediates Activation of ULK Signaling and Transcriptional Regulation of Autophagy Linked to Liver Inflammation and Regeneration

**DOI:** 10.1016/j.celrep.2019.02.013

**Published:** 2019-03-05

**Authors:** Prasun Guha, Richa Tyagi, Sayan Chowdhury, Luke Reilly, Chenglai Fu, Risheng Xu, Adam C. Resnick, Solomon H. Snyder

**Affiliations:** 1 The Solomon H. Snyder Department of Neuroscience, Johns Hopkins University School of Medicine, Baltimore, MD 21205, USA; 2 Department of Psychiatry and Behavioral Sciences, Johns Hopkins University School of Medicine, Baltimore, MD 21205, USA; 3 Department of Pharmacology and Molecular Sciences, Johns Hopkins University School of Medicine, Baltimore, MD 21205, USA; 4 Children’s Hospital of Philadelphia, Colket Translational Research Building, 3501 Civic Center Blvd., Philadelphia, PA 19104-4399, USA; 5 Lead Contact

## Abstract

Autophagy plays a broad role in health and disease. Here, we show that inositol polyphosphate multikinase (IPMK) is a prominent physiological determinant of autophagy and is critical for liver inflammation and regeneration. Deletion of IPMK diminishes autophagy in cell lines and mouse liver. Regulation of autophagy by IPMK does not require catalytic activity. Two signaling axes, IPMK-AMPK-Sirt-1 and IPMK-AMPK-ULK1, appear to mediate the influence of IPMK on autophagy. IPMK enhances autophagy-related transcription by stimulating AMPK-depen-dent Sirt-1 activation, which mediates the deacetylation of histone 4 lysine 16. Furthermore, direct binding of IPMK to ULK and AMPK forms a ternary complex that facilitates AMPK-dependent ULK phosphorylation. Deletion of IPMK in cell lines and intact mice virtually abolishes lipophagy, promotes liver damage as well as inflammation, and impairs hepatocyte regeneration. Thus, targeting IPMK may afford therapeutic benefits in disabilities that depend on autophagy and lipophagy—specifically, in liver inflammation and regeneration.

## In Brief

IPMK is a physiological determinant of autophagy and is critical in liver inflammation. Two signaling axes, IPMK-AMPK-Sirt-1 and IPMK-AMPK-ULK1, appear to mediate the influence of IPMK on autophagy. Deletion of IPMK impairs lipophagy and hepatocyte regeneration.

## Graphical Abstract

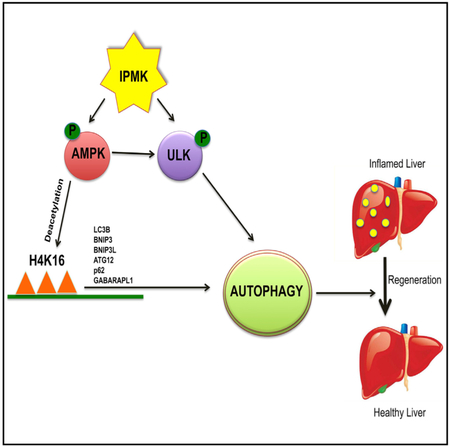

## INTRODUCTION

Autophagy occurs at a basal rate in most cells, eliminating protein aggregates and damaged organelles to maintain cytoplasmic homeostasis. Autophagy can also lead to cell death (Guha et al., 2016) and plays a role in neurodegenerative diseases as well as malignant transformation (Kaur and Debnath, 2015; Galluzzi et al., 2016). Diverse families of genes regulating the autophagic process have been delineated, but how autophagy affects their signaling remains unclear.

Inositol polyphosphates are major signaling molecules generated by a family of inositol phosphate kinases that successively phosphorylate the inositol ring, leading to the formation of inositol hexakisphosphate (IP6) as well as lesser phosphorylated derivatives. IP6, in turn, is phosphorylated to generate inositol pyrophosphates; specifically, one or two isomers of IP7 and IP8 (Maag et al., 2011). Inositol polyphosphate multikinase (IPMK) physiologically generates IP4 and IP5 (Maag et al., 2011). In anon-catalytic fashion, IPMK influences diverse cellular processes, functioning as a co-activator for p53, CREB, p300 (CBP), and serum response factor (SRF) and regulating immediate-early gene transcription (Kim et al., 2011a, 2013; Xu et al., 2013). As one of its kinase-independent activities, IPMK stabilizes the mTORC1 complex (Kim et al., 2011a). IPMK is also a physiological phosphatidylinositol 3-kinase (PI3K), with activity that leads to Akt phosphorylation (Maag et al., 2011). Deletion of IPMK is embryonic lethal in mice, indicating the importance of this enzyme in biology (Maag et al., 2011).

Interactions between IPMK and autophagy have been reported. In yeast, deletion of IPMK leads to virtual abolition of autophagy as well as mitophagy (Taylor et al., 2012). IPMK appears to regulate autophagy genes as well as their link to ULK kinase. Thus, deletion of IPMK markedly reduces transcription of autophagy-associated genes and decreases activation of ULK as well as downstream autophagy signaling. In the present study, we delineate mechanisms whereby IPMK mediates diverse components of autophagy, for which IPMK appears to be a major physiological determinant.

## RESULT

### IPMK Is Essential for Autophagy

**Figure 1.**
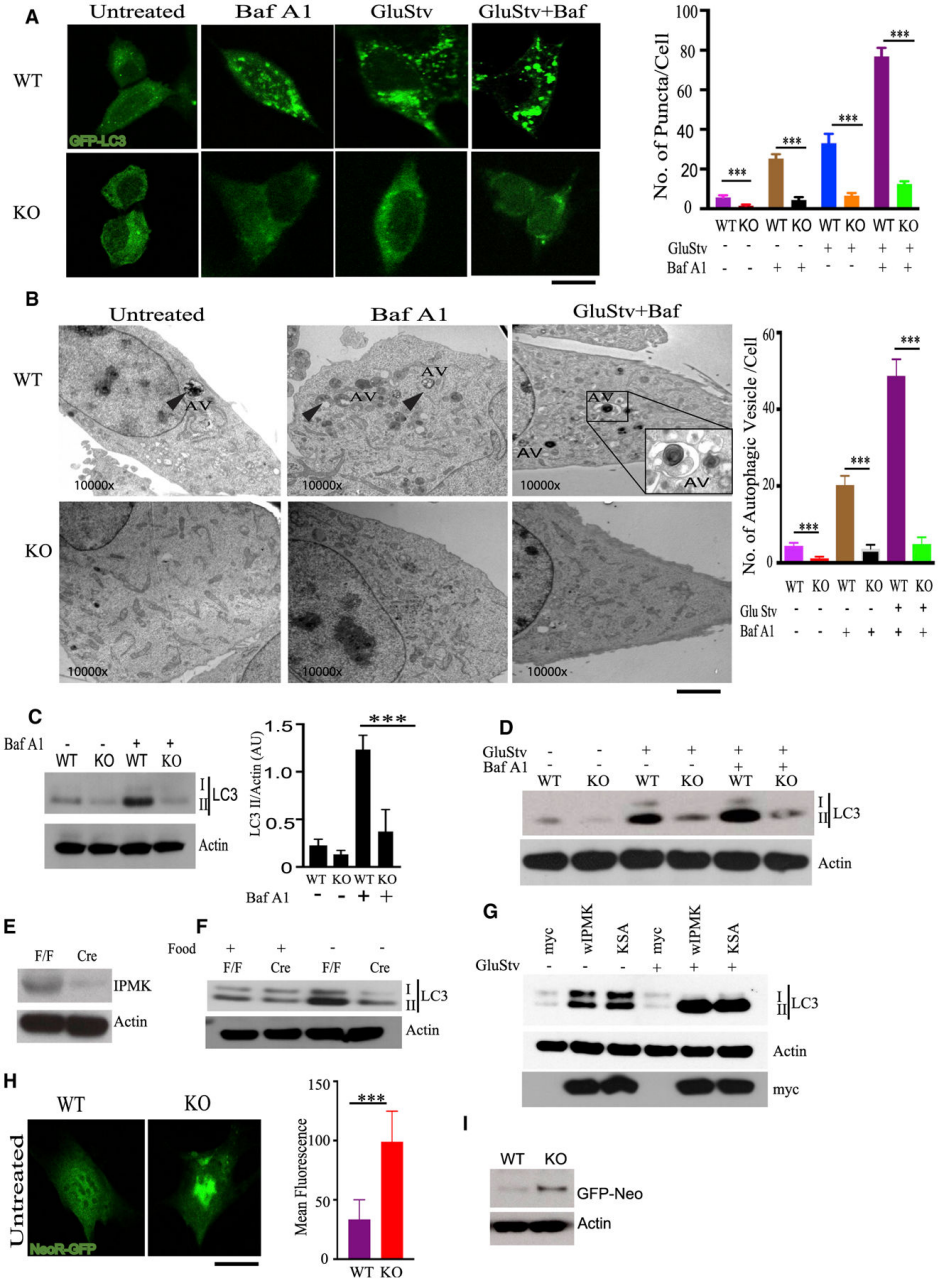
IPMK Is Required for Autophagy (A) IPMK wild-type (WT) and (KO) MEFs were stably transfected with GFP-LC3. Cells were subjected to Baf A1 (100 nM), glucose starvation (GluStv), and GluStv + Baf A1 (100 nM). GFP-LC3 puncta were analyzed using confocal microscopy. Scale bar, 20 μM. The bar chart shows numbers of puncta per cell. (B) Transmission electron microscopy (TEM) of WT and KO MEFs subjected to different treatments. AV, autophagic vacuole. Scale bar, 2 μM. Autophagic vacuoles per cell are shown as bar diagrams. (C) The basal level of autophagy was evaluated by western blotting LC3 with Baf A1 (100 nM). The bar chart depicts the densitometric relative value of LC3-II and Actin. n = 3, ***p < 0.001. (D) LC3 western blot to check autophagic flux under GluStv and GluStv + Baf A1. (E) Western blot of the IPMK level in F/F and IPMK-deleted (Cre) livers. (F) LC3 western blot in F/F and Cre (IPMK KO) livers and after 24 h of food starvation. (G) IPMK KO MEFs were stably transfected with empty vector (myc), IPMK WT (wIPMK) myc, and kinase-dead myc (KSA) IPMK. Autophagy was evaluated by western blotting LC3 II levels with and without GluStv. Baf A1 (100 nM) was used to analyze autophagic flux. (H) NeoR-GFP was transiently transfected in WT and KO MEFs. Twenty-four hours after transfection, cells were analyzed using confocal microscopy. Scale bar, 20 μM. The bar chart depicts the mean fluorescence level of GFP. (I) The amount of NeoR-GFP was analyzed by western blotting NeoR-GFP in WT and KO MEF. Data are means ± SD.

To investigate the roles of IPMK in autophagy, we generated immortalized IPMK wild-type (WT)/knockout (KO) MEFs (mouse embryonic fibroblasts) (Maag et al., 2011). IPMK KO MEFs displayed impaired spreading, a well-established feature of autophagy suppression (Sharifi et al., 2016; Figure S1A). We monitored autophagy by quantifying LC3 puncta, which correspond to autophagic vesicles (Klionsky et al., 2016). WT and KO MEFs stably expressing GFP-LC3 were exposed to bafilomycin A1 (Baf A1) to analyze basal autophagic flux (Klionsky et al., 2016), which was markedly diminished in KO MEFs (Figure 1A). Glucose starvation, employed as a stimulus for autophagy, significantly enhanced autophagic flux, with the increase reduced about 70% in IPMK KO MEFs (Figure 1A).

We validated the confocal data using transmission electron microscopy (TEM) (Klionsky et al., 2016). Under basal conditions, IPMK KO MEFs experienced an almost 70% loss of double-membrane autophagic vesicles. Glucose starvation markedly enhanced the numbers of autophagosomes in WT MEFs, which were greatly diminished in KO preparations (Figure 1B).

Global IPMK deletion in mice is embryonic lethal (Maag et al., 2011). Accordingly, we created liver-specific conditional KOs of IPMK by crossing flox/flox IPMK mice with albumin-Cre (alb-Cre) mice (STAR Methods; Figure S1B). Electron microscopy analysis of 24-h-starved mouse liver tissue sections revealed autophagic vesicle formation in IPMK floxed/floxed (F/F) (WT) liver, which was markedly diminished in IPMK F/F-AlbCre liver (KO) (Figure S1C), confirming the role of IPMK in regulation of autophagy.

Phosphatidylethanol-conjugated ATG8/LC3 is a widely used biochemical marker of autophagy (Sharifi et al., 2016). LC3-I is non-lipidated, whereas LC3-II is the lipidated form. Levels of LC3-II are employed as markers of autophagosome formation and accumulation (Klionsky et al., 2016). To evaluate basal autophagic flux, cells were treated with Baf A1. Deletion of IPMK virtually abolished LC3-II levels, implying a major role of IPMK in determining basal levels of autophagy (Figure 1C). We also stimulated autophagy through glucose starvation for 8 h. Deletion of IPMK markedly suppressed LC3 lipidation in glucose-starved MEFs (Figure 1D). We employed 24 h of food deprivation, a process that induced robust LC3-II expression in the livers of F/F mice (IPMK F/F). In contrast, IPMK-deleted KO mice (IPMK F/F-AlbCre) failed to express LC3-II (Figures 1E and 1F).

To ensure that the findings with glucose starvation can be generalized to other autophagic stimuli, we evaluated H_2_O_2_ treatment, which is well-known to elicit autophagy (He et al., 2017). IPMK deletion abolished LC3-II enhancement associated with H_2_O_2_ treatment (Figure S1D).

We extended our findings to a different cell type. Using small hairpin RNA (shRNA), we stably knocked down IPMK in 786-0 renal cancer cells (Figure S1E). Knockdown of IPMK with shRNA clone 3 in 786-0 cells significantly reduced enhancement of LC3-II levels by glucose starvation (Figure S1F).

IPMK possesses distinct inositol phosphate kinase activity and PI3K activity (Figure S1G; Maag et al., 2011). To ascertain the importance of IPMK’s catalytic activity in regulating autophagy, we overexpressed IPMK WT or IPMK-KSA (IPMK K129A/S235A), which is kinase-dead (devoid of inositol triphosphate [IP_3_] and phosphatidylinositol 3-phosphate [PIP3] kinase activity) and verified their enzymatic activity through inositol profiling (Figure S1H). We attempted to reverse the decreased autophagy associated with IPMK deletion by rescuing IPMK KO MEFs with WT or kinase-dead IPMK mutants (Figure 1G). Ki-nase-dead IPMK mutants rescued the loss of LC3-II in IPMK KO cells as effectively as IPMK WT (Figure 1G). Thus, the catalytic activity of IPMK is not required for its enhancement of autophagy.

Removal of exclusive autophagic substrates (not proteasomal substrates) provides an independent way to analyze autophagy. Neomycinphosphotransferase II (NeoR) is an exclusive autophagic substrate (Nimmerjahn et al., 2003; Chauhan et al., 2013; Yang et al., 2011). As established earlier, NeoR-GFP degradation is completely inhibited by autophagic inhibitors like 3-methyladenine (3-MA) but does not respond to inhibitors of proteasomal degradation. Inhibition of autophagy leads to accumulation of NeoR-GFP, resulting in enhanced GFP fluorescence (Nimmerjahn et al., 2003; Chauhan et al., 2013; Yang et al., 2011). We transfected WT and KO MEFs with NeoR-GFP plasmids, and 24 h after transfection we analyzed sequestration of NeoR-GFP using confocal imaging and western blotting. Under basal conditions, WT MEFs displayed uniform cytoplasmic and nuclear fluorescence. However, in KO cells, brightly fluorescent protein aggregates were evident in nuclear proximal regions, with a greatly enhanced mean fluorescence intensity (Figure 1H). Western blots showed stronger NeoR-GFP bands in KO than in WT cells (Figure 1I), implying defects in basal autophagy.

### Deletion of IPMK Profoundly Suppresses Transcription of Autophagy-Related Genes by Deactivating Sirtuin 1

We showed previously that IPMK can function as a transcriptional co-activator and control transcription of immediate-early genes (Xu et al., 2013). IPMK has also been found to control the transcriptional activity of HDAC (Watson et al., 2012; Bosch and Saiardi, 2012). Arg82, the yeast homolog of IPMK, controls transcription of a set of genes important for arginine metabolism (Bosch and Saiardi, 2012).

**Figure 2.**
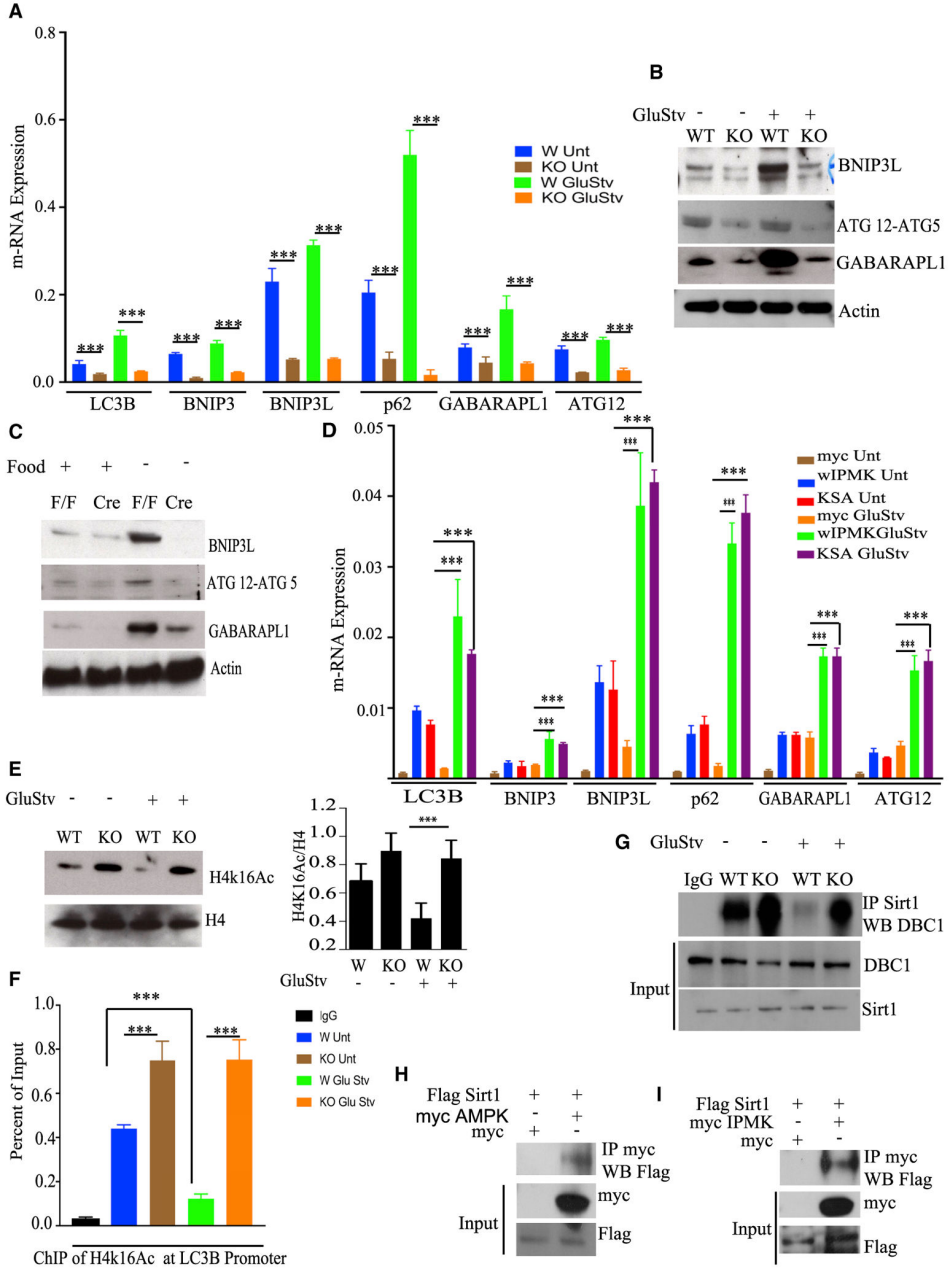
IPMK Enhances Transcription of Autophagy-Related Genes (A) qPCR analysis of LC3B, BNIP3, BNIP3L, p62, GABARAPL1, and ATG12 in IPMK WT and KO MEFs after GluStv. (B) Western blot of BNIP3L, ATG12, and GABARAPL1 in IPMK WT and KO MEFs after GluStv. (C) Western blot of BNIP3L, ATG12, and GABARAPL1 in F/F and Cre (IPMK KO) mouse livers after 24 h of food starvation. (D) IPMK KO MEFs were stably transfected with empty vector (myc), wIPMK myc, and kinase-dead myc (KSA) IPMK. Shown is a qPCR analysis of LC3B, BNIP3, BNIP3L, p62, GABARAPL1, and ATG12 in myc, wIPMK, and KSA MEFs. (E) Western blot analysis of histone 4 lysine 16 acetylation (H4k16ac). (F) Chromatin immunoprecipitation of H4k16 at the LC3B promoter from WT and KO MEFs. n = 3, ***p < 0.001. (G) Immunoprecipitation of Sirt-1 and western blot of DBC1 before and after GluStv in WT and KO MEFs. (H) HEK293 cells were transfected with FLAG Sirt-1 and myc AMPK or empty vector of myc. Immunoprecipitation of myc was followed by FLAG western blotting. (I) HEK293 cells were transfected with FLAG Sirt-1 and myc IPMK and empty vector of myc. Immunoprecipitation of myc was followed by FLAG western blotting. Data are means ± SD.

To analyze IPMK’s role as a transcriptional regulator, we performed qPCR of 6 autophagy-related genes. We selected LC3B and GABARAPL1, which facilitate elongation and closure of autophagic vesicles (Joachim et al., 2015; Slobodkin and Elazar, 2013); BNIP3 and BNIP3L, which help initiate macro-autophagy and selective forms of autophagy, such as mitophagy (Zhang and Ney, 2009; Quinsay et al., 2010); ATG12, a ubiquitin-like protein involved in autophagic vesicle formation (Fader and Colombo, 2009); and P62 (sqstm1), an adaptor protein that recruits cargo to autophagic vesicles (Kaur and Debnath, 2015). Deletion of IPMK in MEFs markedly impaired mRNA expression of these genes in untreated preparations and under glucose starvation (Figure 2A). Furthermore, we performed western blotting of BNIP3L, ATG12-ATG5, and GABARAPL1 to confirm the qPCR data. The protein levels of the above genes were induced by glucose starvation and virtually abolished in IPMK KO MEFs (Figure 2B).

We extended our findings to 786-0 cells, in which deletion of IPMK markedly reduced the protein levels of BNIP3L, ATG12, and GABARAPL1 (Figure S2A). *In vivo*, western blot analysis of mouse liver samples after 24 h of food starvation showed significant increases in BNIP3L, ATG12-ATG5, and GABRAPL1 in IPMK F/F mice; they were markedly diminished in IPMK F/F-AlbCre mice (KO) (Figure 2C). We also examined MEFs treated with H_2_O_2_. Within 1 h of H_2_O_2_ exposure, we observed a substantial increase in the mRNA levels of LC3B, BNIP3, BNIP3L, p62, GABARAPL1, and ATG12; they were markedly decreased in IPMK KO MEFs (Figure S2B).

The decreased mRNA expression of LC3B, BNIP3, BNIP3L, p62, GABARAPL1, and ATG12 was rescued by both the WT and kinase-dead forms of IPMK (Figure 2D). Thus, the regulation of transcription of autophagy-related genes by IPMK is independent of its kinase activity.

IPMK can regulate both histone acetylation and deacetylation, depending on specific stimuli (Watson et al., 2012; Xu et al., 2013). Induction of autophagy requires downregulation of histone H4 lysine 16 acetylation (H4k16ac) (Fullgrabe et al., 2013; Sakamaki et al., 2017). To ascertain whether IPMK regulates autophagy through deacetylation of h4K16, we monitored the levels of H4k16ac. Glucose starvation in WT cells markedly downregulated H4k16ac, whereas deletion of IPMK completely suppressed deacetylation of h4K16 (Figure 2E). Further, chromatin immunoprecipitation (ChIP) analysis of H4k16ac at the LC3B promoter confirmed starvation-induced loss of h4K16 acetylation in WT cells, with KO levels virtually unchanged (Figure 2F).

Sirtuin 1(Sirt-1) is an important h4K16 deacetylase that regulates starvation-induced autophagy (Sakamaki and Ryan, 2017; Sakamaki et al., 2017; Berger and Sassone-Corsi, 2016). Sirt-1 is activated by nutrient deprivation via its dissociation from its inhibitory binding partner Deleted in Breast Cancer Protein 1 (DBC1) (Kim et al., 2008). AMP-activated protein kinase (AMPK), a kinase activated by nutrient starvation (Kim et al., 2011b), stimulates Sirt-1 activation by dissociating DBC1 from Sirt-1 (Sakamaki et al., 2017). AMPK phosphorylation at Thr172, which is required for its activation and is markedly increased in nutrient and food starvation (Bang et al., 2014), was significantly diminished in IPMK KO cells (MEFs) and liver tissue (Figure S2C). Treatment with 5-aminoimidazole-4-carboxamide ribonucleotide (AICAR), a cell-permeable AMPK stimulant, enhanced AMPK phosphorylation in WT MEFs but strikingly less in IPMK KO MEFs (Figure S2C). Co-immunoprecipitation of Sirt-1/DBC1 revealed binding of both proteins in WT and KO MEFs under basal conditions. Glucose starvation in WT cells abolished Sirt-1/DBC1 binding, whereas, in KO cells, binding was unchanged (Figure 2G).

We wanted to know whether AMPK could physically interact with Sirt-1. Overexpressed AMPK can bind to overexpressed Sirt-1 (Figure 2H). Intriguingly, endogenous AMPK could bind to endogenous Sirt-1 independent of glucose starvation (Figure S2D). Deletion of IPMK diminished the endogenous Sirt-1/AMPK interaction (Figure S2D). Overexpressed IPMK also immunoprecipitated overexpressed and endogenous Sirt-1 in HEK293 cells (Figure 2I; Figure S2E).

Collectively, the data above detail a signaling cascade whereby IPMK helps to activate AMPK, and binding of IPMK and AMPK to Sirt 1 facilitates dissociation of Sirt-1 from DBC1, stimulating Sirt-1 activation. Activated Sirt-1 further enhances deacetylation of H4K16 and transcription of autophagy-related genes (Figure S2F).

### IPMK Mediates AMPK-Dependent ULK Phosphorylation

**Figure 3.**
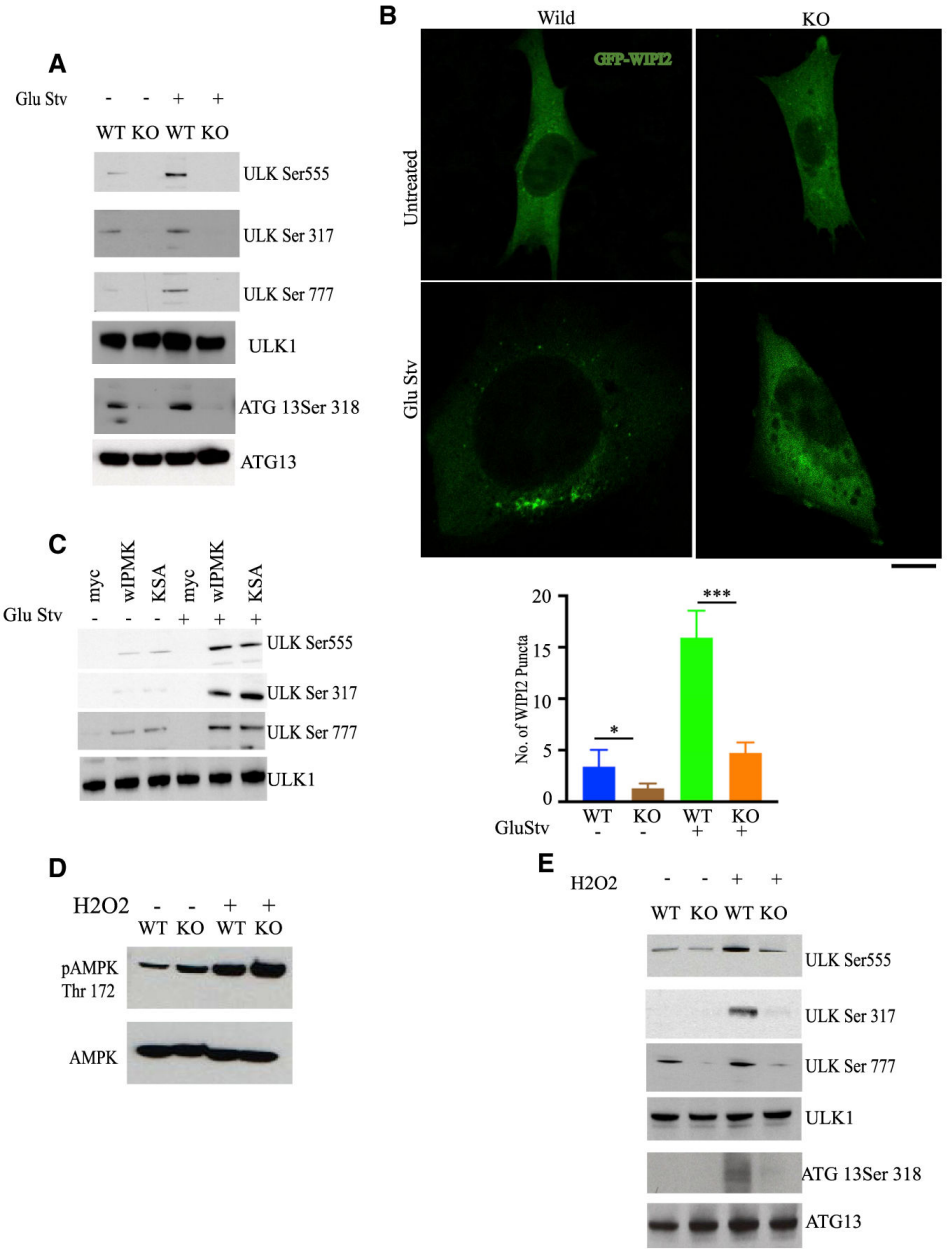
IPMK Regulates Autophagy through ULK Phosphorylation (A) IPMK WT and KO MEFs were subjected to GluStv, followed by immunoblotting of phosphorylated ULK1 at serines 555, 317, and 777. The ATG 13 phosphorylation status indicates the ULK1 activation level. (B) IPMK WT and KO MEFs were transiently transfected with GFP-WIPI2. Confocal analysis of WIPI2 puncta in WT and KO MEFs under untreated and glucose-starved conditions was performed. Scale bar, 20 μM. The bar chart depicts the numbers of WIPI2 puncta per cell. (C) Followed by GluStv Immunoblot of phosphorylated ULK1 at serines 555, 317, and 777 in IPMK KO MEFs (myc) complemented with wIPMK or the kinase-dead form (KSA). (D) Phosphorylation of AMPK in WT and KO MEFs after H_2_O_2_ (500 μM) treatment. (E) IPMK WT and KO MEFs were treated with H_2_O_2_ (500 μM), followed by immunoblotting of phosphorylated ULK1 at serines 555, 317, and 777. The ATG 13 phosphorylation status indicates the ULK1 activation level. Data are means ± SD.

AMPK may also influence autophagy through ULK phosphorylation. ULK(Unc-51-like autophagy-activating kinase) is one of the earliest mediators of autophagy (Itakura and Mizushima, 2010). Kim et al. (2011b) and Egan et al. (2011) provided evidence that AMPK and the mechanistic target of rapamycin (mTOR) regulate initiation of autophagy by phosphorylating ULK. mTOR inhibits ULK activity by phosphorylating the enzyme at serine 757. Under conditions of nutrient stress, AMPK enhances autophagy by phosphorylating raptor, thereby inhibiting mTORC1 and activating phosphorylation of ULK at serines 555, 317, and 777. We agree with the previous finding because deletion of AMPK (alpha 1/2) (double KO [DKO]) impaired ULK phosphorylation at serines 555, 317, and 777 (Figure S3A). Because IPMK regulates AMPK activation, we explored the influence of IPMK on phosphorylation of ULK at the AMPK sites S-555/317/777 (Figure 3A). IPMK deletion abolished AMPK-associated phosphorylation of ULK at S-555/317/777 under glucose starvation.

Because IPMK deletion abolished the activating phosphorylation events on ULK, one might anticipate decreases in ULK-dependent phosphorylation with IPMK deficit. Accordingly, we monitored phosphorylation of the ULK substrate ATG 13 (Egan et al., 2015; Orsi et al., 2012; Figure 3A). IPMK deletion abolished phosphorylation of ATG 13 under glucose starvation.

We next assessed WIPI2 (WD repeat domain phosphoinositide-interacting protein 2) punctum formation, which correlates with the amount of PtdIns(3)P produced by the class III PtdIns(3) kinase complex (Russell et al., 2013; Dooley et al., 2014) and reflects activation of the autophagy-specific PtdIns(3) kinase VPS34. Activation of ULK elicits phosphorylation of VPS34 and stimulates its PtdIns(3) kinase activity (Dooley et al., 2014). Confocal imaging revealed that IPMK depletion of glucose-starved MEFs suppressed WIPI2 punctum formation but not protein levels (Figure 3B).

We rescued the lost ULK phosphorylation of IPMK-deleted cells by overexpressing IPMK WT and the kinase-dead form, which restored these phosphorylation events (Figure 3C).

We showed that regulation of ULK phosphorylation by IPMK occurs in intact animals (Figure S3B). Food deprivation markedly augmented ULK-S-555 phosphorylation, which was substantially reduced in the livers of IPMK-deleted mice.

IPMK can regulate AMPK phosphorylation under nutrient starvation (Figure S2C), which might mediate the influence of IPMK on ULK. However, H_2_O_2_ can directly induce AMPK phosphorylation by oxidative modification of the AMPKα subunit (Zmijewski et al., 2010). Consistent with this model, in IPMK-deleted MEFs, H_2_O_2_-stimulated levels of phos-pho-AMPK were comparable with the WT (Figure 3D). Interestingly, ULK phosphorylation at the AMPK site after H_2_O_2_ treatment was still significantly reduced in IPMK KO MEFs (Figure 3E). Thus, the loss of ULK phosphorylation in IPMK KOs is not just secondary to any alteration in AMPK phosphorylation.

### IPMK Regulates ULK Phosphorylation by Direct Binding Interactions

**Figure 4.**
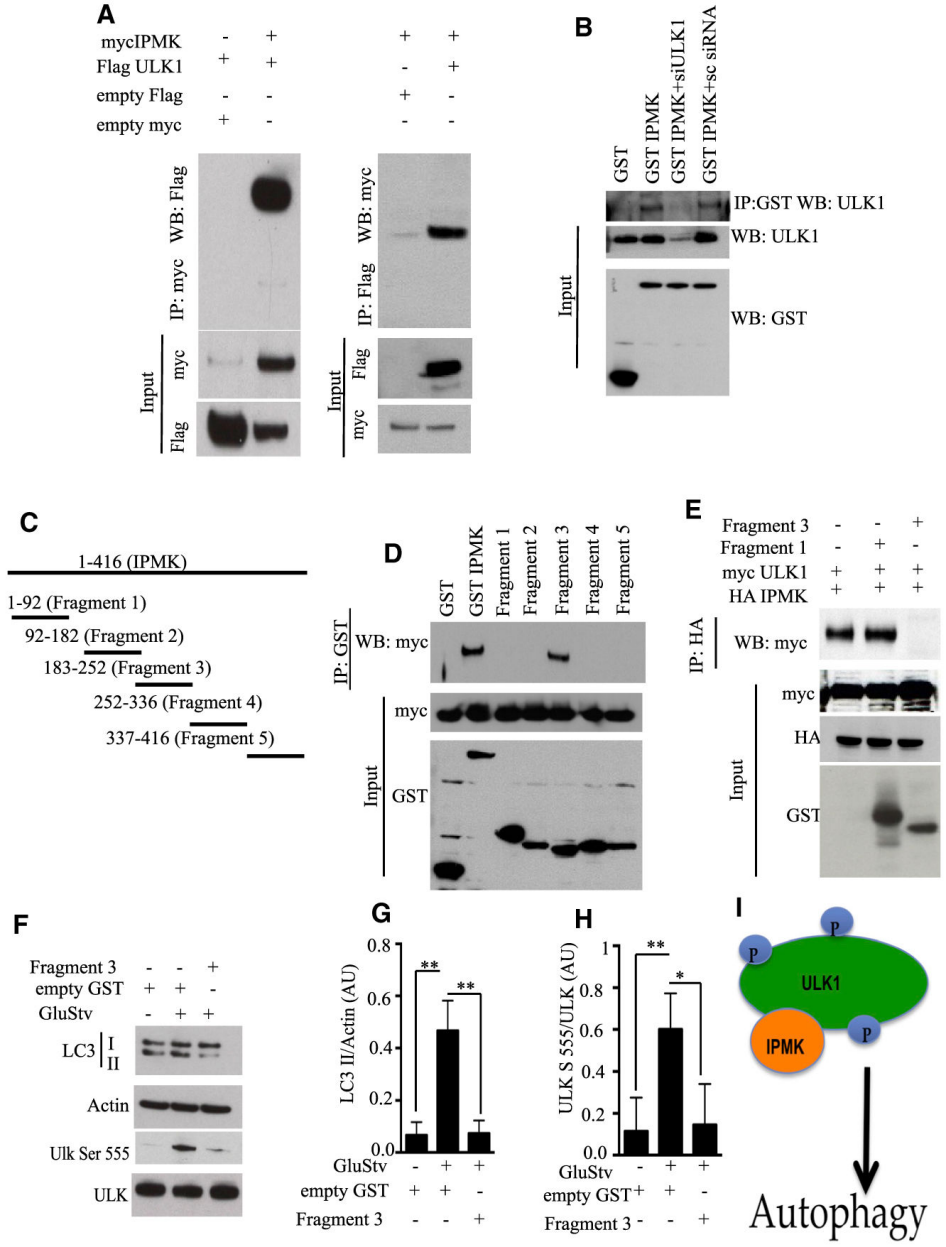
IPMK Regulates ULK Phosphorylation by Direct Binding Interactions (A) Empty myc, empty FLAG, myc IPMK, and FLAG ULK1 were co-transfected in HEK293 cells, and co-immunoprecipitation was performed (n = 4). (B) Glutathione S-transferase (GST), GST IPMK, and GST IPMK in combination with small interfering RNA (siRNA) of ULK1 and scrambled (sc) siRNA were co-transfected in HEK293 cells. Immunoprecipitation of GST was followed by western blotting of endogenous ULK1. (C) Schematic diagram of fragments of IPMK. (D) Different fragments of GST IPMK were cotransfected with myc ULK1. Immunoprecipitation of GST followed by western blot of myc was performed to map IPMK binding to ULK1 (n = 3). (E) Analysis of dominant negatives to assess binding of IPMK to ULK1 (n = 3). (F) Overexpression of an IPMK dominant-negative fragment (fragment 3) in HEK293 cells, followed by GluStv for 6 h. Functional evaluation of the dominant-negative action of fragment 3 was performed by immunoblotting LC3II and ULK serine 555 (n = 3). (G and H) Relative amounts of LC3II (G) and ULK serine 555 (H) are plotted (n = 3); ***p < 0.01, *p < 0.05. Data are means ± SD. (I) Schematic diagram of IPMK binding to ULK1, which facilitates ULK1 phosphorylation and activation of autophagy. Data are means ± SD.

Because IPMK’s regulation of autophagy does not require its kinase activity, we studied direct binding of IPMK to ULK. IPMK bound ULK regardless of whether the pull-down employed ULK or IPMK (Figure 4A). We also showed that endogenous ULK binds IPMK (Figure 4B). The absence of satisfactory antibodies to IPMK precluded evaluation of endogenous IPMK binding interactions. Utilizing *in vitro* systems, we did demonstrate direct binding of ULK and IPMK (Figures S4A and S4B). To facilitate manipulation of the IPMK and ULK system, we mapped sites on IPMK responsible for binding ULK (Figures 4C and 4D). Fragment 3, comprising amino acids 182–252, appeared to be a candidate dominant-negative structure because it substantially inhibited IPMK and ULK binding (Figure 4E). Acting as a dominant-negative fragment, fragment 3 reduced the influence of glucose starvation on LC3-II in HEK293 cells (Figures 4F–4H). In contrast, fragment 1, comprising amino acids 1–92, failed to influence LC3-II levels or ULK phosphorylation (Figure S4C). These findings indicate that binding of IPMK to ULK mediates ULK phosphorylation and autophagy (Figure 4I).

### Direct Binding of IPMK to AMPK Is Required for IPMK’s Influence on Autophagy

**Figure 5.**
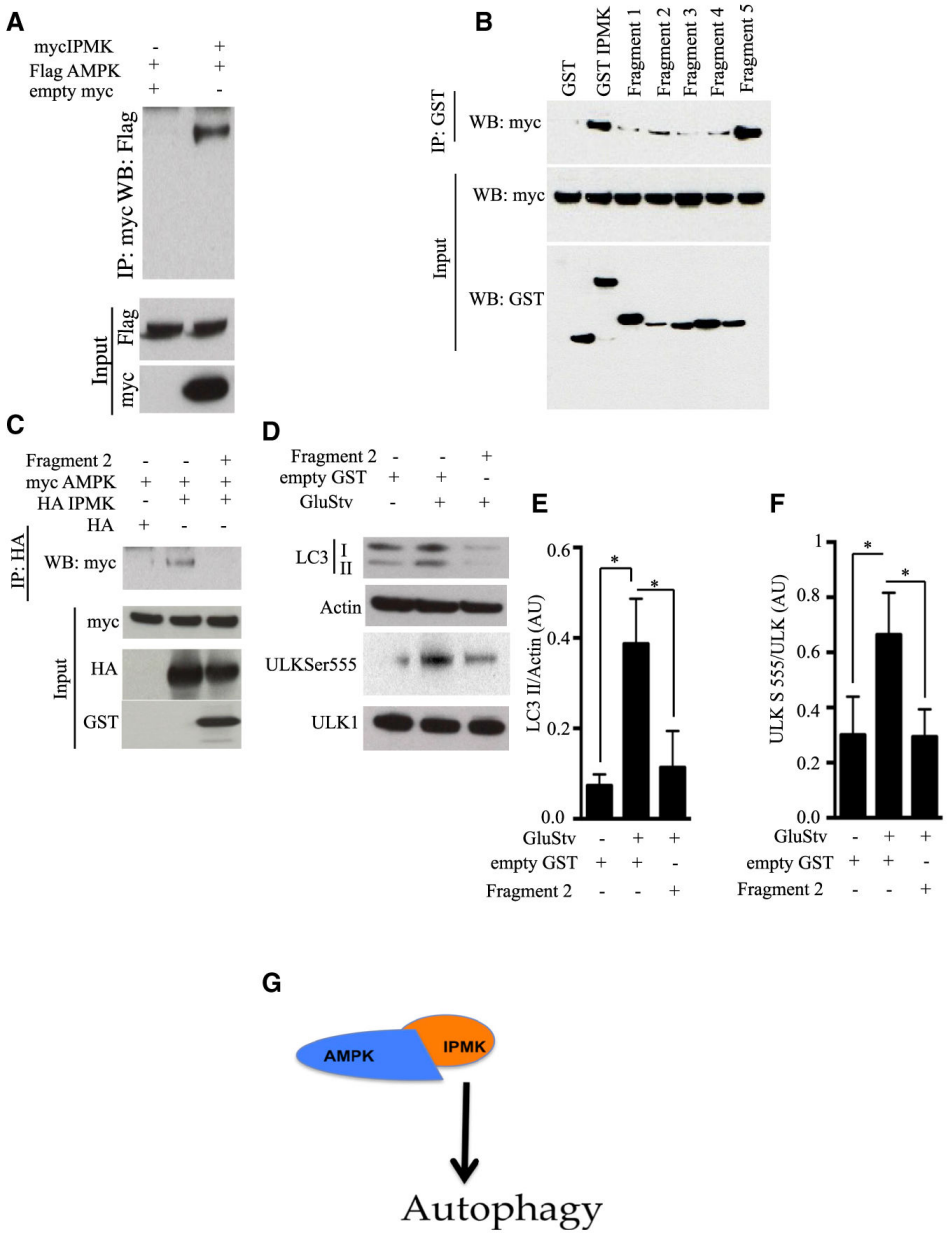
Direct Binding of IPMK to AMPK Is Required for IPMK Influence on Autophagy (A) Empty myc, myc IPMK, and FLAG AMPK were co-transfected in combination in HEK293 cells, and co-immunoprecipitation was performed (n = 4). (B) Different fragments of GST-IPMK were cotransfected with myc AMPK Alpha 2. Immunoprecipitation of GST followed by western blotting of myc was performed to map IPMK binding to AMPK (n = 3). (C) Analysis of dominant negatives for binding of IPMK to AMPK (Kaur and Debnath, 2015). (D) Overexpression of an IPMK dominant-negative fragment (fragment 2) in HEK293 cells, followed by GluStv for 6 h. Functional evaluation of dominant-negative action of fragment 2 was performed by immunoblotting LC3II and ULK serine 555 (n = 3). (E and F) Relative amounts of LC3II (E) and ULK serine 555 (F) are plotted; *p < 0.05. Data are means ± SD. (G) Schematic diagram of IPMK binding to AMPK, facilitating ULK1 phosphorylation and activation of autophagy. Data are means ± SD.

In intact cells, overexpressed IPMK and AMPK bound to each other (Figure 5A). To determine whether binding was direct, we monitored the interactions of the purified IPMK and AMPK proteins (Figures S5A and S5B). We observed substantial direct binding of IPMK and AMPK. We mapped binding sites on IPMK, establishing that the binding is primarily associated with fragment 2, comprising amino acids 92–182 (Figure 5B). Fragment 2 may offer promise as a dominant-negative fragment because it abolished IPMK and AMPK binding (Figure 5C). We employed fragment 2 as a dominant-negative fragment to explore the importance of IPMK in regulating ULK. Overexpressing fragment 2 greatly reduced ULK-S-555 phosphorylation as well as LC3 lipidation (Figures 5D–5F). Although fragment 5 bound AMPK, it failed to serve as a dominant-negative fragment (Figure S5C). Intriguingly, dominantnegative fragment 2 suppressed LC3b gene expression at mRNA levels (Figure S5D). These findings indicate that binding of IPMK to AMPK mediates ULK phosphorylation and autophagy (Figure 5G).

### IPMK Is Essential for AMPK and ULK Interactions

**Figure 6.**
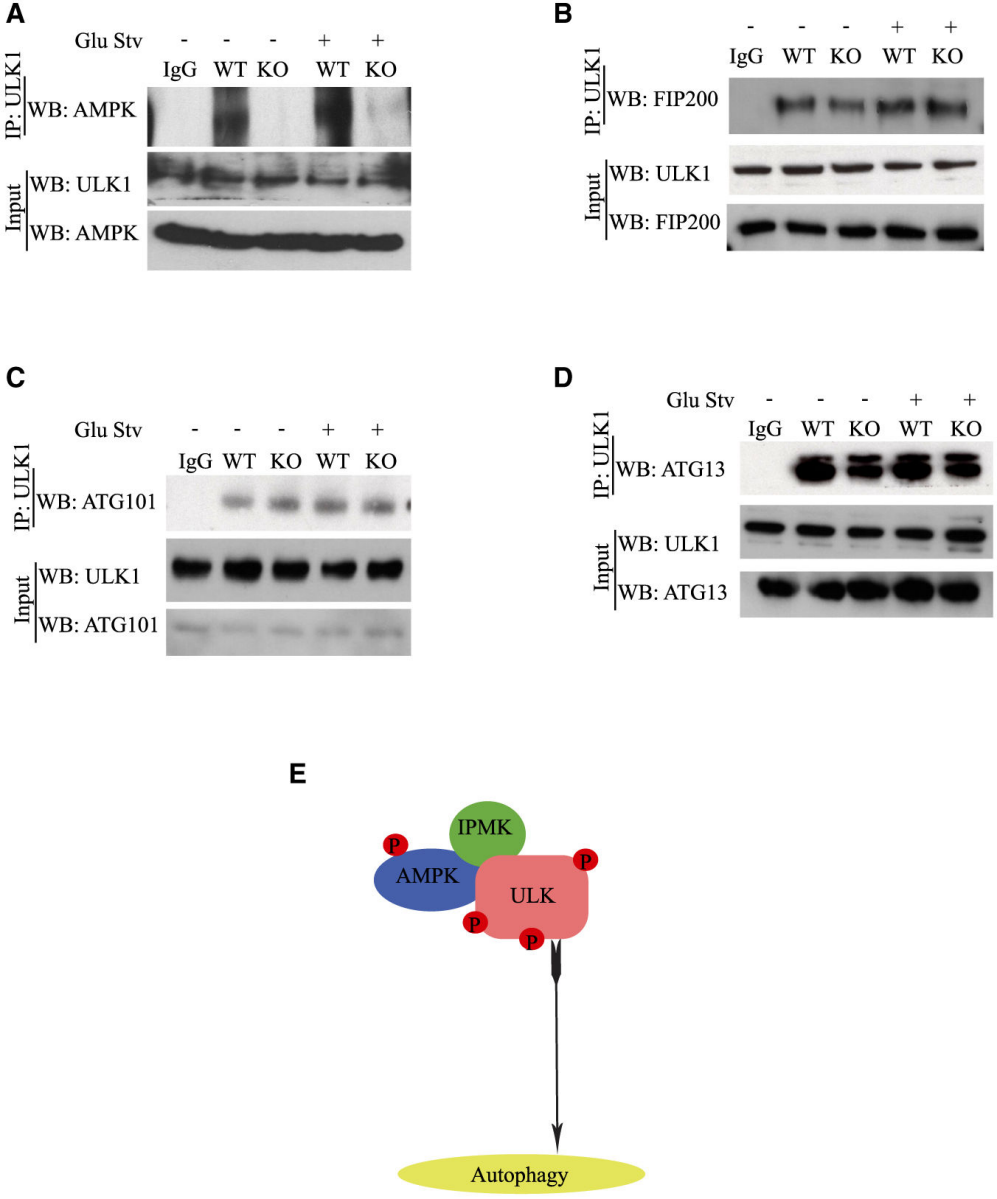
IPMK Is Essential for AMPK and ULK1 Interactions (A–D) IPMK WT and KO MEFs were glucose-starved. The role of IPMK as a scaffold was analyzed by immunoprecipitation of endogenous ULK1 and western blotting of endogenous AMPK (A), FIP200 (B), ATG101 (C), and ATG 13 (D). (E) IPMK interacts with ULK and AMPK to form a ternary complex that facilitates AMPK-dependent ULK phosphorylation.

IPMK is required for binding of ULK and AMPK because their binding was abolished in IPMK KO MEFs with or without glucose starvation (Figure 6A). This action is selective because IPMK deletion did not influence binding of ULK to FIP200 (Figure 6B), ATG101 (Figure 6C), or ATG 13 (Figure 6D). We extended this finding to H_2_O_2_ treatment, which acted the same as glucose starvation (Figures S6A-S6D). We buttressed these conclusions in experiments employing *in vitro* ULK phosphorylation by AMPK. Addition of recombinant human IPMK (hIPMK) (100 ng, 500 ng, and 1 μg) augmented AMPK-dependent ULK phosphorylation (Figure S6E). ULK phosphorylation reached saturation at 500 ng of IPMK. Thus, IPMK is essential for AMPK and ULK interactions and AMPK-dependent ULK phosphorylation (Figure 6E).

### IPMK Is Required for Lipophagy and Regulates Liver Inflammation and Hepatocyte Regeneration

**Figure 7.**
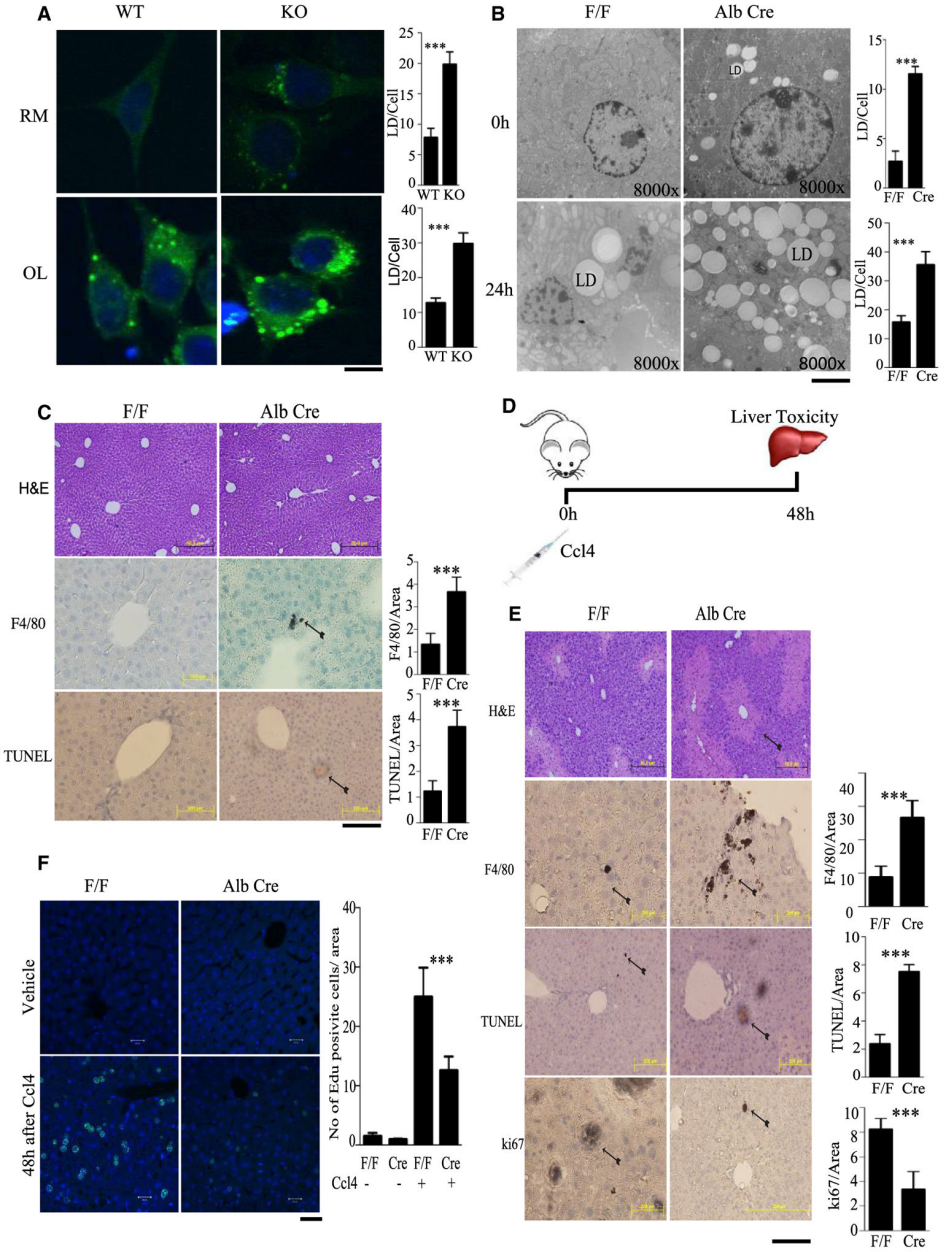
IPMK Is Required for Lipophagy, Cytoprotection, and Cell Regeneration (A) Triglyceride levels in IPMK WT and KO MEFs treated in regular medium (RM) or oleate (OL), measured by staining with boron-dipyrromethene (BODIPY) 493/503. Relative amounts of lipid droplets (LDs) were plotted. ***p < 0.001. Data are means ± SD. Scale bar, 20 μM. (B) IPMK F/F and AlbCre mice were starved for 24 h, and livers were harvested for electron microscopy analysis of lipid droplets in untreated and 0-h- and 24-h-starved mice. Lipid droplets were counted and are represented as bars. ***p < 0.001. Data are means ± SD. Scale bar, 2 μM. (C) Histology of intact livers from IPMK F/F and AlbCre mice. Tissue sections were stained with H&E to check cellular texture and immunostained with F4/80 to monitor inflammatory cells and terminal deoxynucleotidyl transferase dUTP nick end labeling (TUNEL) for apoptosis of hepatocytes. Inflammatory cells and TUNEL-positive cells were counted and are represented as bars. n = 4, ***p < 0.001. Data are means ± SD. Scale bar, 200 μM. (D) Schematics of the experimental protocol. IPMK F/F and AlbCre mice were injected intraperitoneally with a single acute dose (2 μg/g) of Ccl4, followed by liver harvest after 48 h of treatment. (E) Staining of liver sections with H&E, F4/80, TUNEL, and Ki67. Inflammatory cells, TUNEL-positive cells, and Ki67-stained cells were counted and are represented as bars. n = 4, ***p < 0.001. Data are means ± SD. Scale bar, 200 μM. (F) EDU incorporation in vehicle-treated and Ccl4-treated mouse livers. EDU-positive cells were counted and are represented as bars. n = 4. ***p<0.001. Data are means ± SD. Scale bar, 20 μM.

Abundant data implicate IPMK in regulation of autophagy. One form of macroautophagy, called lipophagy, has been shown to contribute to hydrolysis of triacylglycerol stored in cytoplasmic lipid droplets. Accordingly, we evaluated a potential role of IPMK in regulating lipophagy. IPMK-deleted MEFs displayed a doubling of lipid droplets both in regular medium and with oleate treatment, indicating substantial diminution of lipophagy (Figure 7A). Starvation induces hepatic autophagy and increases delivery of free fatty acids (FFAs) from adipose tissue lipolysis to the liver. Electron microscopy and oil red O staining revealed that both under untreated conditions and overnight starvation, the numbers of lipid droplets were substantially increased in F/F-AlbCre (liver-specific IPMK KO) mice compared with F/F (WT) mice (Figure 7B; Figure S7A), indicating impaired lipophagy in IPMK-deleted livers. The effect of IPMK and AMPK signaling on lipid droplet formation was analyzed by overexpressing fragment 2 (dominantnegative for IPMK and AMPK binding). The number of lipid droplets increased significantly in fragment 2 (Figure S7D).

We wondered whether IPMK deficiency affected overall liver function, which we assessed by monitoring the serum levels of alanine-leucine transaminase (ALT), which were unchanged in IPMK KOs and F/F-AlbCre mice (Figure S7B). We examined liver morphology by H&E staining, which was not altered in F/F-AlbCre mice. However, we observed a mild increase in inflammatory cell number in F/F-AlbCre livers as well as a modest enhancement of apoptotic cells (Figure 7C).

We evaluated the response of IPMK-deleted livers (F/F-AlbCre) to cytotoxic insults utilizing carbon tetrachloride (Ccl4) (Figure 7D). Damage was increased substantially in IPMK KO livers (Figure 7E). The damage associated with Ccl4 was especially notable, with increased numbers of inflammatory cells and apoptotic cell profiles as well as serum ALT levels (Figure S7C). These observations indicate that IPMK is cytoprotective. We also monitored liver regeneration following Ccl4 administration (Figures 7E and 7F). We measured Ki67, an index of replicative cell activity, as well as 5-ethynyl-2’-deoxyuridine (EDU) incorporation into regenerating cells. Cell regeneration appeared to decrease by about 50% in IPMK KO livers.

## DISCUSSION

The present study establishes IPMK as a principal determinant of autophagy and of cytoprotection in the liver. Deletion of IPMK abolishes autophagy, monitored in multiple ways, indicating that IPMK is a physiological regulator of autophagy (Figure 1). Catalytic activity of IPMK is not required for this. Mechanistically, IPMK regulates autophagy in two different ways. (1) IPMK influences transcription of autophagy-related genes by regulating H4K16 deacetylation. (2) IPMK mediates AMPK-dependent ULK phosphorylation. We also showed that deletion of IPMK impairs lipophagy in cell lines and intact liver (Figures 7A and 7B). Most importantly, deletion of IPMK augments liver inflammation and impedes hepatocyte regeneration (Figures 7D and 7E).

IPMK KO mice die early in development, around embryonic day 9.5 (E9.5), because of severe growth and morphological defects resembling the phenotype of mice with deletion of major autophagy-regulatory genes, such as Beclin 1, FIP200, and Ambra1 (Maag et al., 2011; Mizushima and Levine, 2010; Seeds et al., 2015). Deletion of IPMK in *Drosophila* substantially impairs adult tissue growth and stability (Seeds et al., 2015). Loss of yeast IPMK (Arg82) retards growth and increases susceptibility to stress-induced death (Szijgyarto et al., 2011). Moreover, deletion of yeast IPMK impairs autophagy and mitophagy (Taylor et al., 2012; Kanki et al., 2009).

How does IPMK influence the autophagic process? IPMK regulates autophagy through AMPK. AMPK initiates autophagy by regulating the transcription of autophagic genes and activating ULK. Nutrient deprivation promotes Sirt-1-mediated deacetylation of H4K16, followed by transcription of autophagy-related genes (Fullgrabe et al., 2013). AMPK enhances dissociation of Sirt-1 from its inhibitor DBC1 and stimulates transcription of autophagy-related genes (Sakamaki et al., 2017). We found that deletion of IPMK markedly diminishes AMPK activation during glucose starvation and impairs AMPK-mediated H4K16 deacetylation (Figure 2). On the other hand, AMPK phosphorylates ULK at serines 555, 317, and 777 and activates autophagy by recruiting the beclin1 complex and activating the class III PI3K VPS34 (Lamb et al., 2013). AMPK-dependent ULK phosphorylation is abolished with deletion of IPMK (Figure 3). It could be anticipated that IPMK influences ULK phosphorylation by activating AMPK. However, with H_2_O_2_ treatment, IPMK-deleted MEFs have increased levels of phospho-AMPK, comparable with the WT (Figure 3F), although ULK phosphorylation at the AMPK site is significantly diminished (Figure 3G). Protein-protein interaction studies confirmed that IPMK acts as a scaffold protein, linking AMPK with ULK (Figures 4, 5, and 6).

In summary, we present two signaling cascades, IPMK-AMPK-H4K16 and IPMK-AMPK-ULK, that regulate transcription and ULK phosphorylation to initiate autophagy (Figure S7E). IPMK positively regulates lipophagy and is also essential for cytoprotection of the liver and hepatocyte regeneration (Figures 7A-7F). Autophagy has been implicated in numerous conditions, such as neurodegenerative diseases, cocaine toxicity (Guha et al., 2016; Galluzzi et al., 2016; Yang et al., 2011), cancer, and obesity (Haim et al., 2015). IPMK’s influence on autophagy may be also a major mediator of these diverse actions.

## STAR★METHODS

### CONTACT FOR REAGENT AND RESOURCE SHARING

Further information and requests for resources and reagents should be directed to and will be fulfilled by the Lead Contact, Solomon H. Snyder MD (ssnyder@jhmi.edu).

### EXPERIMENTAL MODEL AND SUBJECT DETAILS

#### Animals

All protocols were approved by the Johns Hopkins University Animal Care and Use Committee. Mice were housed according to institutional guidelines, in a controlled environment at a temperature of 22°C ± 1°C, under a 12-h dark-light period and provided with standard chow diet and water *ad libitum.* Male and female IPMK F/F, IPMK F/F-Alb Cre, (between 1 and 3-month-old) were used. Specifically, male mice were used for liver regeneration study, electron microscopy and tissue histology. All mice were maintained in 129SV-C57BL/6 mixed background.

#### Cell culture

Mouse embryonic fibroblasts (MEF) and human embryonic kidney HEK293T cells (American Type Culture Collection) were maintained in a humid atmosphere of 95% air and 5% CO2 at 37°C in DMEM 786-0 renal cancer cell was marinated in RPMI-1640 supplemented with 10% FBS, L-glutamine (2 mM), and penicillin (100 units/mL)/streptomycin (100 mg/mL). Retroviral transfection and generation of stably transfected cells:

Retroviral constructs (5) were transiently transfected into a Platinum-E retroviral packaging cell line for 48 h by using Lipofectamine 2000 transfection reagent. The high-titer viral stocks were produced by passing the supernatant using a 0.45 μm pore size-filter. For infection, MEFs were incubated with the viral supernatant in the presence of polybrene (8 μg/mL) for 48 h. Stably infected MEFs were selected with blasticidin (4 μg/mL) for 1-2 weeks. Selected stable cell lines were always maintained in respective medium containing blasticidin (4 μg/mL).

#### Generation of brain specific IPMK knock out mice

IPMK F/F mice were generated as described previously (5). To develop brain specific IPMK KO, IPMKfl/fl mice were crossed with FLP delete (Neomycin) and Nestin Cre driver (brain specific cre driver) mice. FRT sites flanking the neomycin resistance gene facilitate its removal by FLP recombinase,and loxP sites facilitate removal of the targeted exon 6 by Cre recombinase. IPMKfl/fl mice were mated with mice expressing FLP recombinase to excise the neomycin resistance gene to generate IPMKflpped/flpped mice (we refer IPMK F/F in the paper). Homozygous IPMK F/F mice were crossed with the Albumin Cre+/− mice, which mediate excision of floxed alleles in liver, mostly in hepatocytes. Genotyping was performed using a transnetyx genotyping facility. All mice were maintained on a 129SV-C57BL/6 mixed background. Animal care and experimentations were approved by the Johns Hopkins University Animal Care and Use Committee. Mice were housed in a 12-h light/12-h dark cycle, at an ambient temperature of 22°C, and fed standard rodent chow.

### METHOD DETAILS

#### Chemicals, antibodies and reagents

Hydrogen peroxide, bafilomycin, antifade gold mounting medium, FBS, L-glutamine, penicillin /streptomycin, DMEM without glucose, DMEM without sodium pyruvate for H2O2 treatment medium, PVDF membrane, DMSO cell culture grade, Lipofectamine 2000, Lipofectamine 3000 were purchased from Thermo Fisher Scientific. Polyfect was purchased from qiagen. Antibodies for actin, LC3, ATG5, myc, VPS34, ULK ser 317, ULK ser 757, ATG 13, ATG 13pSer 318, Beclin1, Beclin Ser 15, VPS34 Ser 249, AMPK, PAMPK Thr 172,Flag, GST, HA,FIP200, ATG101 were purchased from Cell Signaling Technology. ULK1ser555 and ULK ser 777 were from Millipore. ULK1 for immunoprecipitation was purchased from Sigma. Ulk1 for western blots was purchased from Santacruz Biotechnology. Anti-mouse IPMK was developed in our lab.

#### Cell culture

Mouse embryonic fibroblasts (MEF) and human embryonic kidney HEK293T cells (American Type Culture Collection) were maintained in a humid atmosphere of 95% air and 5% CO2 at 37°C in DMEM supplemented with 10% FBS, L-glutamine (2 mM), and penicillin (100 units/mL)/streptomycin (100 mg/mL). PC12 (ATCC) cells were maintained in DMEM supplemented with 10% (vol/vol) horse serum, 5% (vol/vol) FBS, and 2 mM L-glutamine.

#### Generation of brain specific IPMK knock out mice

IPMK F/F mice were generated as described previously (5). To develop brain specific IPMK KO, IPMKfl/fl mice were crossed with FLP delete (Neomycin) and Nestin Cre driver (brain specific cre driver) mice. FRT sites flanking the neomycin resistance gene facilitate its removal by FLP recombinase,and loxP sites facilitate removal of the targeted exon 6 by Cre recombinase. IPMKfl/fl mice were mated with mice expressing FLP recombinase to excise the neomycin resistance gene to generate IPMKflpped/flpped mice. Homozygous IPMKflpped/flpped mice were crossed with the Albumin Cre+/− mice, which mediate excision of floxed alleles in liver, mostly in hepatocytes. Genotyping was performed using a transnetyx genotyping facility. All mice were maintained on a 129SV-C57BL/6 mixed background. Animal care and experimentations were approved by the Johns Hopkins University Animal Care and Use Committee. Mice were housed in a 12-h light/12-h dark cycle, at an ambient temperature of 22°C, and fed standard rodent chow.

#### Plasmids and recombinant proteins

The following plasmids were from Addgene: pMXs-puroGFP-DFCP1,pCDNA6-myc ULK1 wt, pCDNA3 Flag ULK1, pCDNA3 myc AMPK Alpha 2, pBABE-puro-mCherry-GFP-LC3,and pBABE-puro-GFP-LC3. pCMV-AMPK alpha 2 Flag was from Sinobiologicals. Our lab generated pCMV-IPMK-GST and different fragments tagged with GST, pCMV-myc-IPMK, pCMV-HA-IPMK, pMX-myc, pMX-myc IPMK WT, and pMX-myc KSA. Constitutively active AMPK was a kind gift from Dr. Anne Burnet (Stanford University School of Medicine, Stanford, CA). We purchased recombinant hIPMK from Origene. Recombinant hULK1 and AMPK were purchased from Signalchem.

#### Autophagy activation in mouse liver

Mice (male) were starved overnight followed feeding for 6 h to synchronize the food cycle. Then one group of mice was allowed to feed, while a second group of mice (IPMK F/F and IPMK F/F-Albumin Cre) was starved for 24 h. Starvation of food induced autophagy in liver was analyzed by western blot of LC3-II and ULK ser 555. Each group comprised 3 mice.

#### Activation of autophagy in cells

1× 10^5^Cells (MEF and others) were glucose starved for 8 h followed by lysing them for biochemical analysis(30). Oxidative stress was elicited by treatment with H2O2 (500 uM) for 30 min, which induced substantial autophagy in MEFs and other cell lines. Autophagic flux was analyzed using Baf A1 100 nM to inhibit lysosomal degradation of autophagic vesicles (12). Autophagy was analyzed either by western blot of LC3-II, transmission electron microscopy, or confocal microscopy.

#### Transmission Electron Microscopy (TEM)

MEFs were treated with indicated concentrations of H_2_0_2_, bafilomycin, or by glucose starvation. The cells were fixed in 2.5% glutaraldehyde, 3 mM MgCl2, in 0.1 M sodium cacodylate buffer, pH 7.2, for one h at room temperature. After buffer rinse, samples were post-fixed in 1% osmium tetroxide in buffer (1 h) on ice in the dark. The cells were stained with 2% aqueous uranyl acetate (0.22 mm filtered, 1 h in the dark), dehydrated in a graded series of ethanol solutions and embedded in Eponate 12 (Ted Pella) resin. Samples were polymerized at 37°C for 2-3 days before moving to 60°C overnight. Thin sections, 60 to 90 nm, were cut with a diamond knife on a Reichert-Jung Ultracut E ultramicrotome and picked up with 2×1 mm copper slot grids. Grids were stained with 2% uranyl acetate in 50% methanol and lead citrate at 4°C and observed with a Hitachi 7600 TEM. Images were captured with an AMT CCD XR50 (2K × 2K) camera. Classification and counting of autophagic vacuoles were done by double-blinded independent observers.

To study liver tissue we starved mice for 24 h followed by surgical excision of the liver. Livers were fixed in 2% (wt/vol) paraformaldehyde (freshly prepared from EM grade prill form), 2% (vol/vol) glutaraldehyde, 3 mM MgCl2, in 0.1 M sodium cacodylate buffer, pH 7.2, overnight. Regions of interest were dissected and samples were washed in 0.1 M sodium cacodylate buffer with 3 mM MgCl2 and 3% (wt/vol) sucrose. Samples were postfixed in reduced 2% (wt/vol) osmium tetroxide, 1.6% (wt/vol) potassium ferrocyanide in buffer (2 h) on ice in the dark. Samples were stained with 2% (wt/vol) aqueous uranyl acetate (0.22 μm filtered, 1 h in the dark), dehydrated in a graded series of ethanol propylene oxide solutions, and embedded in Eponate 12 (Ted Pella) resin. Samples were polymerized at 60°C overnight. Thin sections (60–90 nm) were cut with a diamond knife on a Reichert-Jung Ultracut E ultramicrotome and picked up with 2 × 1 mm copper slot grids. Grids were stained with 2% (wt/vol) uranyl acetate in 50% (vol/vol) methanol and lead citrate at 4°C and observed with a Hitachi 7600 TEM. Images were captured with an AMT CCD XR50 (2K × 2K) camera.

#### qPCR analysis

After extracting the total RNA using Gen Elute Mammalian Total RNA miniprep kits (Sigma), and checking its integrity by electrophoresis, the cDNA was synthesized from 1 mg of purified total RNA using Revert Aid H minus first strand cDNA synthesis kit (Fermentas Life Sciences, Ontario,Canada). Expression of mouse and human IPMK, mouse LC3B, mouse BNIP3 and mouse GAPDH was detected using suitably designed Taqman primers (Invitrogen).Other genes such as mouse BNIP3L, P62,GABARAPL1 and ATG12 were determined using primers given in the table. qPCR of these genes was performed using power Sybr green pcr mater mix from Invitrogen. Expression of the designated enzymes was normalized against glyceraldehyde-3-phosphate dehydrogenase (GAPDH) as the internal reference. The experiments were performed (real-time PCR Systems StepOne plus, Applied Biosystems) in triplicate. Data were quantified for the above genes using the comparative Ct method, as described in the Assays-on-Demand Users Manual (Applied Biosystems).

qPCR Primers (Sybr green):

catcgtggagaaggctccta- gabarapl1 (mF)

atacagctggcccatggtag- gabarapl1 (mR)

AACAAAGAAATGGGCTGTGG – ATG12 (mF)

TTGCAGTAATGCAGGACCAG- ATG12 (mR)

CCTCGTCTTCCATCCACAAT- bnip3l (mF)

GTCCCTGCTGGTATGCATCT- bnip3l (mR)

TGGCCACCTCTCTGATAGCT- p62 (mF)

TCATCGTCTCCTCCTGAGCA- p62 (mR)

#### ChIP

Approximately 7×10^6^ cells were fixed with 1% formaldehyde (Sigma-Aldrich, Cat#: F8775) at room temperature for 10 min followed by ChIP using ChIP assay Kit from Millipore-Sigma (17-295). Cells were then harvested and lysed in 500 mL of ChIP lysis Buffer (50 mM Tris-HCl pH 8.0, 5 mM EDTA, 150 mM NaCl, 0.5% Triton X-100, 0.5% SDS, 0.5% NP-40, 1 mM sodium butylate) containing protease inhibitor cocktail. The lysates were subjected to sonication to shear DNA to a length of approximately between 150 and 900 bp. The lysate was then diluted in 1.2 mL of ChIP dilution buffer and incubated with control IgG (Cell Signaling Technology, Cat#: 2729S) or primary antibody H4k16ac (Active Motif 39168) 4C overnight. Then the lysate was incubated with Protein A/ Salmon sperm slurry (provided in kit) for 1 h at 4C. The beads were washed sequentially with wash buffer provided in kit. The immunocomplexes were eluted with 75 mL of elution buffer (1% SDS, 0.1 M NaHCO3) twice at 65C for 30 min. After elution, the cross-link was reversed by adding NaCl and incubated together with Proteinase K (Thermo Fisher Scientific, Cat#: EO0491) overnight at 65C. ChIP DNA was purified using ChIP DNA purification kit (Actif motif 58002). The purified DNA was analyzed on a StepOnePlus using power SYBR Green Master Mix. The results are presented as percentage of input. qPCR analyses were done in triplicate. We used LC3B primers:(F) CATGCCTTGGGACACCAGAT, (R) ACCTTCTTCAAGTGCTGTTTGT (45).

#### Confocal microscopy of autophagy

IPMK WT and KO MEFs were stably transfected with LC3 GFP. IPMK WT and KO cells were transiently transfected with NeoR-GFP and GFP-WIPI2 constructs using lipofectamine 3000. Cells were treated with different conditions and images were captured in a confocal microscope using Zeiss LSM 800. Images were analyzed with Zenlite software. The puncta were counted using Imaris x64 7.7.2 software.The intensity of NeoR-GFP was analyzed using ImageJ.

#### Western blots

Cell lysates were prepared using lysis buffer (150 mM NaCl, 0.5% CHAPS, 0.1% Triton, 0.1% BSA, 1 mM EDTA, protease inhibitors, phosphatase inhibitors). Samples were centrifuged at 14,000 g for 20 min, and the protein concentration of the supernatant was measured. Proteins were resolved by SDS-polyacrylamide gel electrophoresis and transferred to PVDF membranes. The membranes were blocked for 2 h at room temperature in 20 mM Tris-HCl, pH 7.4, 150 mM NaCl, and 0.02% Tween 20 (Tris-buffered saline/Tween 20) containing 3% BSA followed by overnight incubation at 4°C in 1:1000 dilution of the respective antibodies for LC3, actin, ULK1, ULK Ser 555, ULK Ser 317, ULK Ser 777, ULK Ser 757, ATG 13, ATG 13 Ser 318, Bnip3l, ATG12, GABARAPL1, H4k16ac, H4, Sirt-1, DBC1, GFP, AMPK, AMPK Thr 172, FIP200, ATG101, anti-myc, anti-gst, anti-HA, anti-Flag in 3% BSA. The PVDF membrane (Millipore) was washed three times with Tris-buffered saline/Tween-20, incubated with HRP-conjugated secondary antibody (GE health care), and the bands visualized by chemiluminescence (Pierce). The depicted blots are representative replicates selected from at least three experiments. Densitometric analysis was performed using ImageJ software.

#### Immunoprecipitations

pCMV mycIPMK or pCMV myc were cotransfected with pCMV Flag or pCMV ULK1 plasmids into HEK293 cells using polyfect (QIAGEN). Forty-eight hours after transfection, immunoprecipitation of the myc or Flag tagged protein was performed with 500 μg of protein lysates in lysis buffer (150 mM NaCl, 0.5% CHAPS, 0.1% Triton, 0.1% BSA, 1 mM EDTA, protease inhibitors, phosphatase inhibitors) incubated overnight at 4°C EZview myc or Flag beads (Sigma). Beads were pelleted and washed with lysis buffer 3 times, and SDS sample buffer loading dye was added. Immunoprecipitated samples were resolved by polyacrylamide gel electrophoresis. To assess interactions between IPMK and AMPK, HEK293 cells were co-transfected with pCMV mycIPMK or pCMV myc with Flag AMPK followed by immunoprecipitaion of myc using the above-mentioned protocol. In the same way, different IPMK gst fragments were co-transfected either with myc ULK1 or myc AMPK to map the IPMK binding site.

To analyze Sirt-1/AMPK and IPMK/Sirt-1 interaction we overexpressed myc Ampk and Flag Sirt in one reaction myc IPMK and Flag Sirt-1 with emty myc as control.We immunoprecipiated myc using EZview myc beads and western blot flag to check the interaction.

#### *In vitro* AMPK mediated ULK phosphorylation

*In Vitro* assay was performed as previously reported (30).Recombinant GST-hUlk1 protein (ThermoFisher) 500 ng was incubated with 10 ng of recombinant purified AMPK complex (Signal Chem) in kinase assay buffer (20 mM HEPES at pH 7.4, 1 mM EGTA, 0.4 mM EDTA, 5 mM MgCl2 and 0.05 mM DTT) supplemented with 0.2 mM AMP and 0.1 mM cold ATP, for 20 min. Recombinant myc-human IPMK was added to the kinase reaction (100 ng, 500 ng and 1 ug). After the reaction, western blot of ULK S 777 was performed to test the importance of IPMK.

#### Mice Liver toxicity and regeneration study

IPMK F/F and F/F-AlbCre mice were treated acutely with carbon tetrachloride (Ccl4) 2 ul/g. 48 h after treatment serum was collected from blood through cardiac puncture followed by analysis of liver specific serum chemistry for alanine-leucine transaminase (ALT). Liver tissue was fixed in 4% formalin followed by sectioning at 5 μm thickness. Sections were stained with hematoxylin and eosin (H&E),and immunostained with F4/80 (biorad), Ki67 (Abcam) and TUNEL staining followed by light microscopy. To study liver regeneration mice were intraperitoneally injected with 100 μg/g Edu 2h before harvesting liver. Further liver sections were stained with Click-it Edu staining kit (Thermofisher) followed with imaging by confocal microscopy.

#### Analysis of lipophagy

IPMK WT and KO MEFs were cultured in regular medium (RM) or oleate (OL) 0.25mM for 24h in serum free medium followed by staining lipid droplets with BODIPY 493/503 dye as per manufacturer’s protocol (Thermofisher). To study accumulation of lipid droplets in mice liver, mice were either maintained on regular diet or starved overnight, liver tissue was either processed for transmission electron microscopy or tissue samples were cryosectioned for oil red o (ORO) of lipid droplets in liver as per manufacturer’s protocol, abcam.

#### Endogenous immunoprecipitation analysis

To analyze binding of IPMK to endogenous ULK1, gst IPMK was transfected in HEK293 cells. Forty-eight h after transfection, immunoprecipitation of the gst tagged IPMK was performed with 500 μg of protein lysates in lysis buffer (150 mM NaCl, 0.5% CHAPS, 0.1% Triton, 0.1% BSA, 1 mM EDTA, protease inhibitors, phosphatase inhibitors) incubated overnight at 4°C EZview gst beads (Sigma). Beads were pelleted and washed with lysis buffer 3 times, and SDS sample buffer loading dye was added. Immunoprecipitated samples were resolved by polyacrylamide gel electrophoresis followed by western blotting of ULK1 using ULK1 specific antibody (Santacruz Biotechnology). Immunoprecipitation of endogenous ULK1 was further confirmed using siRNA analysis (Santacruz Biotechnology). For DBC1/Sirt-1 interaction studies we immunoprecipitated endogenous Sirt-1 using Sirt-1 specific antibody and western blot with DBC1. We immunoprecipitated endogenous ULK1 using ULK1 antibody and western blot for AMPK, FIP200, ATG101, and ATG 13 from IPMK WT and KO MEF to analyze endogenous interaction of these proteins with ULK.

#### *In vitro* binding assay

Recombinant myc IPMK (Origene) was co-incubated with either recombinant gst ULK1 (Signalchem) or His AMPK (Signalchem) in lysis buffer and the complex maintained for 30 min at 4°C. After the addition of myc beads incubation was continued for an additional 15 min and washed 3 times with ice cold lysis buffer. SDS sample buffer was added. Binding was confirmed by western blotting of gst or His. The purity of recombinant proteins was confirmed by resolving single bands in NuPAGE protein gels that were stained with Simply Blue Safe Stain (Invitrogen).

#### Transfections

##### a. Retroviral transfection and generation of stably transfected cells:

Retroviral constructs (5) were transiently transfected into a Platinum-E retroviral packaging cell line for 48 h by using Lipofectamine 2000 transfection reagent. The high-titer viral stocks were produced by passing the supernatant using a 0.45 μm pore size-filter. For infection, MEFs were incubated with the viral supernatant in the presence of polybrene (8 μg/mL) for 48 h. Stably infected MEFs were selected with blasticidin (4 μg/mL) for 1-2 weeks. Selected stable cell lines were always maintained in respective medium containing blasticidin (4 μg/mL).

##### b. Transient Polyfect and lipofectamine 3000 transfection:

HEK293 cells were transiently transfected with polyfectamine (QIAGEN) using manufacturer’s protocol. MEF cells were transiently transfected with lipofectamine 3000 using manufacturer’s protocol.

##### Intracellular inositol content profiling

MEFs were plated at a density of 250,000 cells per 60 mm plate, then labeled with 60 μCi (1 Ci = 37 GBq) [3H]myo-inositol (PerkinElmer) in conventional cell culture media for three days. To extract soluble inositol phosphates, cell pellets were suspended in 300 μL of ice-cold 0.6 M perchloric acid buffer (0.1 mg/mL IP6, 2 mM EDTA) and incubated on ice for 1 min. Ninety μL of 1 M potassium carbonate with 5 mM EDTA were added and incubated on ice for 1 h. Extracts were centrifuged at 12,000 rpm for 15 min. The supernatant was collected and analyzed by HPLC using a Partisphere SAX column (Whatman Inc.). The column was eluted with a gradient generated by mixing Buffer A (1 mM EDTA) and Buffer B (Buffer A plus 1.3 M (NH4)2HPO4, pH 3.8 with H3PO4). The 1 mL fractions were collected and counted using 5 mL of Ultima-Flo AP mixture (PerkinElmer).

#### Lentiviral Transduction

786-0 human renal cancer cell was transduced with shRNA particles (Sigma) to knock down IPMK and selected cells using puromycin (0.5 micro gram/ ml) antibiotic.

### QUANTIFICATION AND STATISTICAL ANALYSIS

Error bars in the figures represent standard error of the mean and number of experiments is indicated by n in figure legends. n indicates animals employed for the experiment or times an experiment was performed. Specifics are indicated in the figure legends. Statistical significance (two-tails) was tested with Student’s T-Test for two groups or one-way ANOVA for multiple groups with similar size. The differences were considered significant when p < 0.05. All the statistical analysis was performed with Prism 7 program (GraphPad).

## Supplementary Material

1

2

## Figures and Tables

**Table:** KEY RESOURCES TABLE

REAGENT or RESOURCE	SOURCE	IDENTIFIER
Antibodies		
Actin	Cell Signaling	4967; RRID: AB_330288
LC3B	Cell Signaling	2775; RRID: AB_915950
Atg5	Cell Signaling	2630; RRID: AB_2062340
myc	Cell Signaling	2276; RRID: AB_331783
VPS34	Cell Signaling	3811; RRID: AB_2062856
ULKS317	Cell Signaling	12753; RRID: AB_2687883
ULK S 757	Cell Signaling	6888; RRID: AB_10829226
Beclin	Cell Signaling	3738; RRID: AB_490837
Beclin S 15	Cell Signaling	84966
VPS34 S 249	Cell Signaling	13857
AMPK	Cell Signaling	2532; RRID: AB_330331
AMPK Thr 172	Cell Signaling	2535; RRID: AB_331250
Flag	Cell Signaling	8146; RRID: AB_10950495
GST	Cell Signaling	2622; RRID: AB_331670
HA	Cell Signaling	2367; RRID: AB_10691311
DBC1	Cell Signaling	5693; RRID: AB_10706910
GFP	Cell Signaling	2555; RRID: AB_10692764
IgG control	Cell Signaling	2729; RRID: AB_1031062
ATG 13	Genetex	GTX123970
FIP200	Genetex	GTX107387; RRID: AB_10730495
ATG101	Genetex	GTX31415
Bnip3	Genetex	GTX111902; RRID: AB_1949753
ATG12	Genetex	GTX124181; RRID: AB_11171947
GABARAPL1	Genetex	GTX132664
BNIP3L	Genetex	GTX111876; RRID: AB_2036357
Human IPMK	Genetex	GTX104954; RRID: AB_1950594
ULK1 for IP	Sigma	A7481; RRID: AB_1840703
HRP- ANTI MOUSE	GE Health care	NA931V; RRID: AB_772210
HRP-ANTI RABBIT	GE Health care	GENA934; RRID: AB_2722659
ULK S 555	MILLIPORE	ABC124; RRID: AB_11205237
ULK S 777	MILLIPORE	ABC213
Sirt-1	Santacruz Biotechnology	Sc74465; RRID: AB_1229462
ULK1 for WB	Santacruz Biotechnolo	Sc33182; RRID: AB_2214706
H4k16ac	Active Motif	39168; RRID: AB_2636968
H4	Active Motif	61300; RRID: AB_2650524
Mouse specific Anti-IPMK	Lab generated	NA
F4/80	Biorad	MCA497RT; RRID: AB_1102558
Ki67	Abcam	ab16667; RRID: AB_302459
Biological Samples		
pMXs-puroGFP-DFCP1	Addgene	38269
pCDNA6-myc ULK1 wt (Plasmid)	Addgene	27629
pCDNA3 Flag ULK1 (Plasmid)	Addgene	27636
pCDNA3 myc AMPK Alpha 2 (Plasmid)	Addgene	15991
pBABE-puro-GFP-LC3 (Plasmid)	Addgene	22405
pCMV-AMPK alpha 2 Flag (Plasmid) pCMV mycIPMK (plasmid)	Sinobiologicals	HG10394-CF
pCMV Flag	Snyder lab	NA
Chemicals, Peptides, and Recombinant Proteins		
Recombinant hIPMK	Origene	TP309343
Recombinant hULK1	Signalchem	U01-11G
Recombinant purified AMPK complex	Signal Chem	P47-10H-05
Critical Commercial Assays		
Gen Elute Mammalian Total RNA miniprep Kit	Sigma	RTN10
Revert Aid H minus first strand cDNA synthesis kit	Fermentas Life Sciences	K1631
Sybr green PCR mater mix	Invitrogen	4309155
ChIP Assay kit	Millipore-Sigma	415
ChIP DNA purification kit	Active Motif	58002
Click-it Edu Staining Kit	Thermofisher	C10337
Chemicals, Peptides, and Recombinant Proteins		
Recombinant hIPMK	Origene	TP309343
Recombinant hULK1	Signalchem	U01-11G
Recombinant purified AMPK complex	Signal Chem	P47-10H-05
FBS	Sigma	F2442
L-glutamine	Thermo Fischer Scientific	25030081
Penicillin	Thermo Fischer Scientific	10378016
DMEM (w/o glucose)	Thermo Fischer Scientific	11966025
DMEM (w/o sodium pyruvate)	Thermo Fischer Scientific	11965092
PVDF Membrane	MilliporeSigma	IPVH00010
Lipofectamine 2000	Thermo Fischer Scientific	11668019
Lipofectamine 3000	Thermo Fischer Scientific	L3000015
Polyfect	QIAGEN	301105
CCl4	Sigma	270652
Paraformaldehyde 4% Solution in PBS	Santacruz Biotechnology	30525-89-4
Oleate	Sigma	O1008-1G
[3H]myo-inositol	PerkinElmer	NET1168001MC
1% Formaldehyde	Sigma-Aldrich	F8775
Ezview myc beads	Sigma	E6654
Flag beads	Sigma	F2426
Hematoxylin	Sigma	1092490500
TumorTACS *In Situ* Apoptosis Detection Kit	R&D Systems	4815-30-K
Ezview GST beads	Sigma	E6406
BIODIPY 493/503	Thermofisher	D3922
Simply Blue Safe Stain	Invitrogen	LC6060
Recombinant hIPMK	Origene	TP309343
Recombinant hULK1	Signalchem	U01-11G
Recombinant purified AMPK complex	Signal Chem	P47-10H-05
Experimental Models: Cell Lines		
Mouse embryonic fibroblast	Snyder lab	N/A
786-0 human renal cancer cell	ATCC	ATCC-CRL-1932
Hunan embryonic kidney cell	ATCC	ATCC-CRL-1573
Experimental Models: Organisms/Strains		
IPMK F/F mice129SV- C57BL/6 mixed background.	Ozgene	Custom developed
Albumin Cre miceC57BL/6J	Jackson laboratory	3574
Oligonucleotides		
catcgtggagaaggctccta- gabarapl1 (mF)	Invitrogen	N/A
atacagctggcccatggtag- gabarapl1 (mR)	Invitrogen	N/A
AACAAAGAAATGGGCTGTGG – ATG12 (mF)	Invitrogen	N/A
TTGCAGTAATGCAGGACCAG- ATG12 (mR)	Invitrogen	N/A
CCTCGTCTTCCATCCACAAT- bnip3l (mF)	Invitrogen	N/A
GTCCCTGCTGGTATGCATCT- bnip3l (mR)	Invitrogen	N/A
TGGCCACCTCTCTGATAGCT- p62 (mF)	Invitrogen	N/A
TCATCGTCTCCTCCTGAGCA- p62 (mR)	Invitrogen	N/A
Human shIPMK	Sigma	TRCN0000196885
Human shIPMK	Sigma	TRCN0000219804
Human shIPMK	Sigma	TRCN0000196360
Scrambled shRNA	Sigma	SHC016V
mBECLIN1	Taqman Fisher	Mm01265461
mBNIP3	Taqman Fisher	Mm01275600
mSQSTM1	Taqman Fisher	Mm00448091
hIPMK	Taqman Fisher	Hs00852670
h18S	Taqman Fisher	Hs99999901
Software and Algorithms		
Imaris x64 9.0.2	BITPLANE	https://www.bitplane.com/
ZEN lite	Carl Zeiss	https://www.zeiss.com/microscopy/int/downloads.html?vaURL=www.zeiss.com/microscopy/int/downloads/zen.html
ImageJ	NIH IMAGEJ	https://imagej.nih.gov/ij/
Graphpad prism 8	Graphpad prism	https://www.graphpad.com/scientific-software/prism/
Illustrator CC	Adobe	https://www.adobe.com/products/illustrator.html
Photoshop CC	Adobe	https://www.adobe.com/products/photoshop.html

## Highlights

- IPMK is a physiological determinant of autophagy and critical for liver inflammation
- IPMK-AMPK-Sirt-1 and IPMK-AMPK-ULK1 mediate the influence of IPMK on autophagy
- Deletion of IPMK impairs lipophagy and hepatocyte regeneration
