# Supplementary material for: IPMK Mediates Activation of ULK Signaling and Transcriptional Regulation of Autophagy Linked to Liver Inflammation and Regeneration
Source: Cell Rep. Author manuscript; Available in PMC 2019 May 1. (PMC6494083; doi:10.1016/j.celrep.2019.02.013)
Supplement: 1 [file NIHMS1523265-supplement-1.pdf]

Cell Reports, Volume *26*

Supplemental Information

IPMK Mediates Activation of ULK Signaling and Transcriptional Regulation of Autophagy Linked to Liver Inflammation and Regeneration

Prasun Guha, Richa Tyagi, Sayan Chowdhury, Luke Reilly, Chenglai Fu, Risheng Xu, Adam C. Resnick, and Solomon H. Snyder

Figure S 1

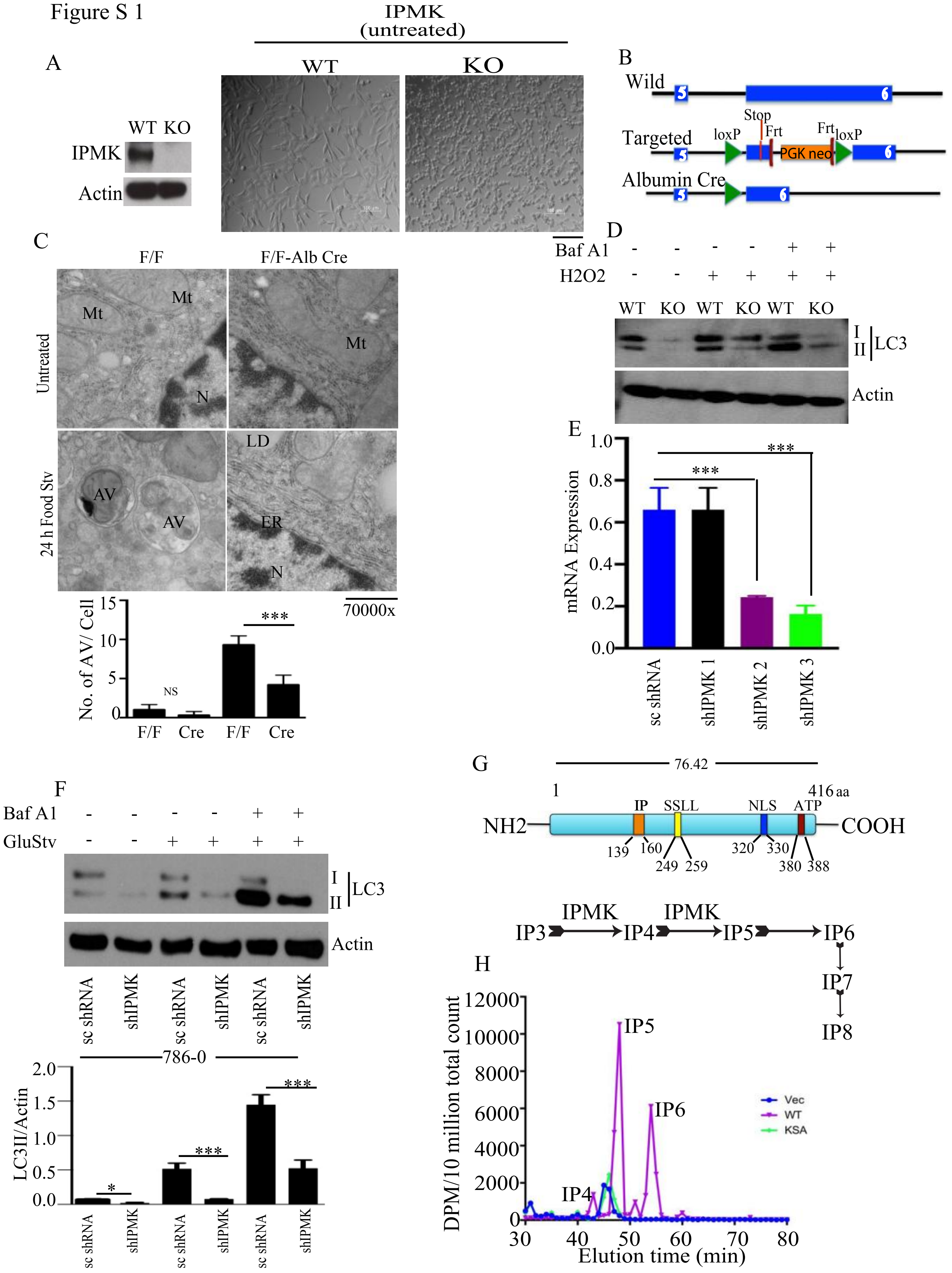

IPMK
(untreated)
A
WT KO
IPMK
Actin
WT
KO
B
Wild
Targeted
loxP
Stop
Frt
PGK neo
Frt
loxP
Albumin Cre
C
F/F
F/F-Alb Cre
Untreated
24 h Food Stv
Mt
N
LD
AV
ER
70000x
No. of AV/ Cell
NS
***
F/F Cre F/F Cre
D
Baf A1
H2O2
WT KO WT KO WT KO
I
II
LC3
Actin
E
mRNA Expression
***
***
sc shRNA
shIPMK 1
shIPMK 2
shIPMK 3
F
Baf A1
GluStv
I
II
LC3
Actin
sc shRNA
shIPMK
786-0
LC3II/Actin
*
***
***
G
76.42
1
416 aa
NH2
COOH
IP
SSLL
NLS
ATP
139 160 249 259 320 330 380 388
IPMK
IPMK
IP3
IP4
IP5
IP6
IP7
IP8
H
DPM/10 million total count
Elution time (min)
IP4
IP5
IP6
Vec
WT
KSA

Figure S1. **IPMK is required for autophagy, Related to Figure 1.**

(A) Western blot of IPMK in W and KO MEF. Phase contrast microscopy of W and KO MEFs. Scale bar 100uM. (B) Schematic diagram for generation of conditional IPMK deletion in mice liver. (C) TEM analysis of F/F and F/F-AlbCre (IPMK KO) liver section with and without food for 24 h. Mt- mitochondria, AV- autophagic vacuole, LD- lipid droplet, N- nucleus, and ER- endoplasmic reticulum. Scale bar 500nM. (D) LC3 western blot to study autophagic flux in IPMK W and KO MEF after H2O2 (500uM) and H2O2+BafA1 treatment for 1h. (E) qPCR analysis of IPMK mRNA expression after shRNA treatment in 786-0 renal cancer cell line. (F) LC3 western blot to study autophagic flux in stably transfected scrambled shRNA and shIPMK (shIPMK3) treated 786-0 renal cancer cells. (G) Schematic map of IPMK protein. Wild type human IPMK is a 76.42-kb gene consisting of 6 exons and 5 introns encoding a 416 amino acid protein with at least 4 structural domains including an inositol phosphate (IP, orange) binding site, Ser-Ser-Leu-Leu catalytically important domain (SSLL, yellow), NLS (nuclear localization signal, blue), and adenosine triphosphate (ATP) binding site (magenta). IPMK catalyzes conversion of IP3 to IP4 and IP5. (H) HPLC analysis of inositol phosphate levels in IPMK KO rescued by vector control (KO + myc), wild type IPMK (KO + WT), kinase dead IPMK (KO + KSA). Data are means ± SD.

Figure S 2

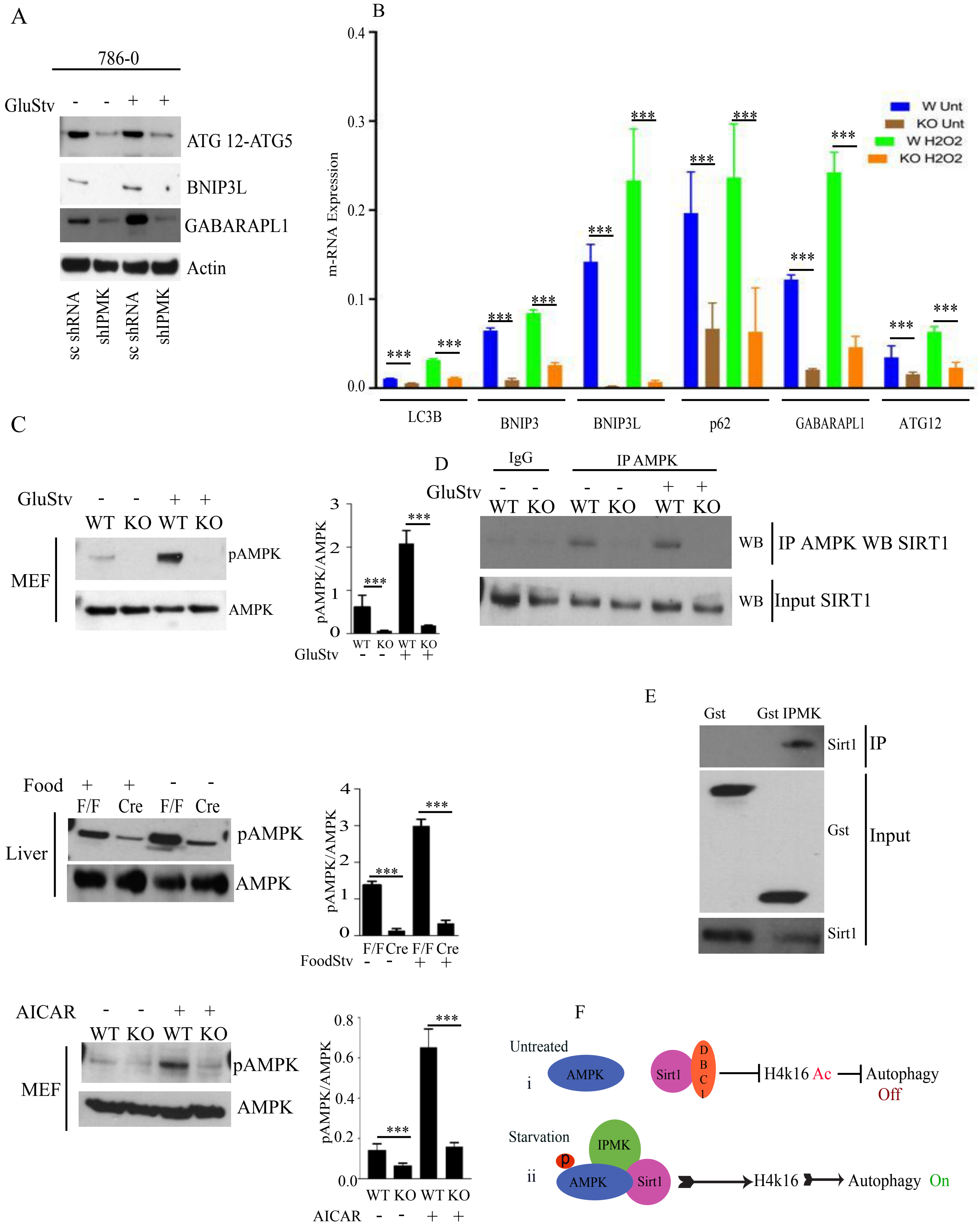

A
786-0
GluStv - - + +
ATG 12-ATG5
BNIP3L
GABARAPL1
Actin
sc shRNA
shIPMK
sc shRNA
shIPMK
B
m-RNA Expression
0.4
0.3
0.2
0.1
0.0
W Unt
KO Unt
W H2O2
KO H2O2
***
LC3B
BNIP3
BNIP3L
p62
GABARAPL1
ATG12
C
GluStv - - + +
WT KO WT KO
MEF
pAMPK
AMPK
pAMPK/AMPK
WT KO WT KO
GluStv - - + +
D
IgG
IP AMPK
GluStv - - - - + +
WT KO WT KO WT KO
WB
IP AMPK WB SIRT1
WB
Input SIRT1
Food + + - -
F/F Cre F/F Cre
Liver
pAMPK
AMPK
pAMPK/AMPK
F/F Cre F/F Cre
FoodStv - - + +
E
Gst
Gst IPMK
Sirt1
IP
Gst
Input
Sirt1
AICAR - - + +
WT KO WT KO
MEF
pAMPK
AMPK
pAMPK/AMPK
WT KO WT KO
AICAR - - + +
F
Untreated
i
AMPK
Sirt1
DBC1
H4k16 Ac
Autophagy
Off
Starvation
ii
IPMK
p
AMPK
Sirt1
H4k16
Autophagy On

Figure S2. IPMK enhances transcription of autophagy related genes, Related to Figure 2.

(A) Western blot of BNIP3L, ATG12 and GABARAPL1 in 786-0 cells stably transfected with scrambled shRNA and shIPMK. (B) qPCR analysis of LC3B, BNIP3, BNIP3L, p62, GABARAPL1, and ATG12 in H2O2 (500uM) treated W/KO MEF. (C) Western blot of phosphoAMPK at Thr 172 in AICAR treated W/KO MEFs and liver tissue from mice. (D) Immunoprecipitation of endogenous AMPK and western blot of endogenous Sirt1. (E) Immunoprecipitation of GST IPMK and western blot of endogenous Sirt1. (F) Schematic diagram of IPMK mediated Sirt1 activation and transcriptional regulation. In untreated cells Sirt1 activity is down-regulated by direct binding of its repressor DBC1. IPMK and AMPK can interact with Sirt1. During glucose starvation IPMK enhances activation of AMPK which enables DBC1 to dissociate from Sirt1 followed by deacetylation of H4K16 and activation of autophagy related gene transcription. Data are means ± SD.

Figure S 3

A

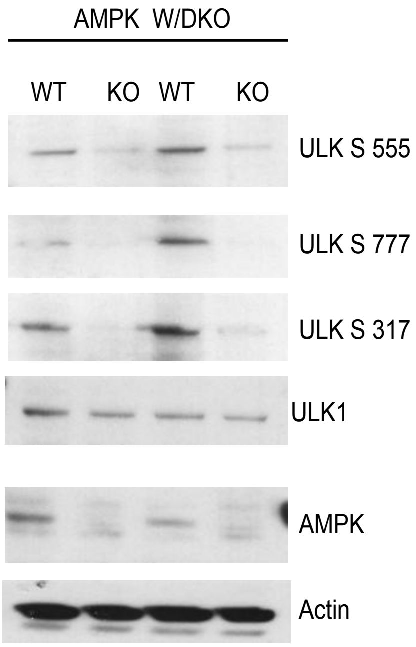
AMPK W/DKO
WT
KO
WT
KO
ULK S 555
ULK S 777
ULK S 317
ULK1
AMPK
Actin

B

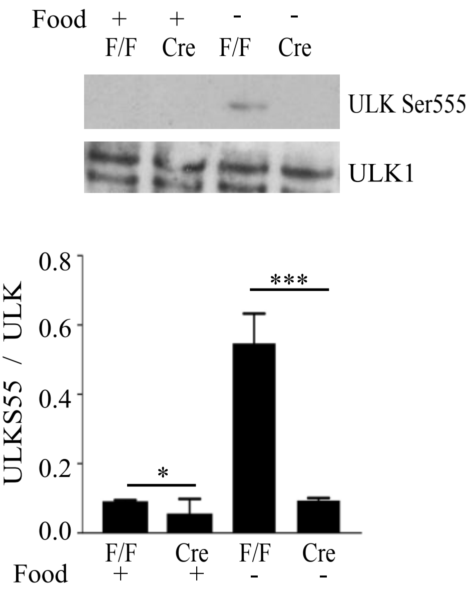
Food + + - -
F/F Cre F/F Cre
ULK Ser555
ULK1
0.8
0.6
0.4
0.2
0.0
ULKS55 / ULK
*
***
F/F Cre F/F Cre
Food + + - -

Figure S3. IPMK regulates autophagy through ULK phosphorylation, Related to Figure 3.

(A) Immunoblot of ULK ser 555, 777, 317 from AMPK double knock (alpha 1/2) out MEFs. (B) Immunoblot of ULK ser 555 in Flox/Flox and IPMK KO (AlbCre) mice liver after 24 h food deprivation.

Figure S 4

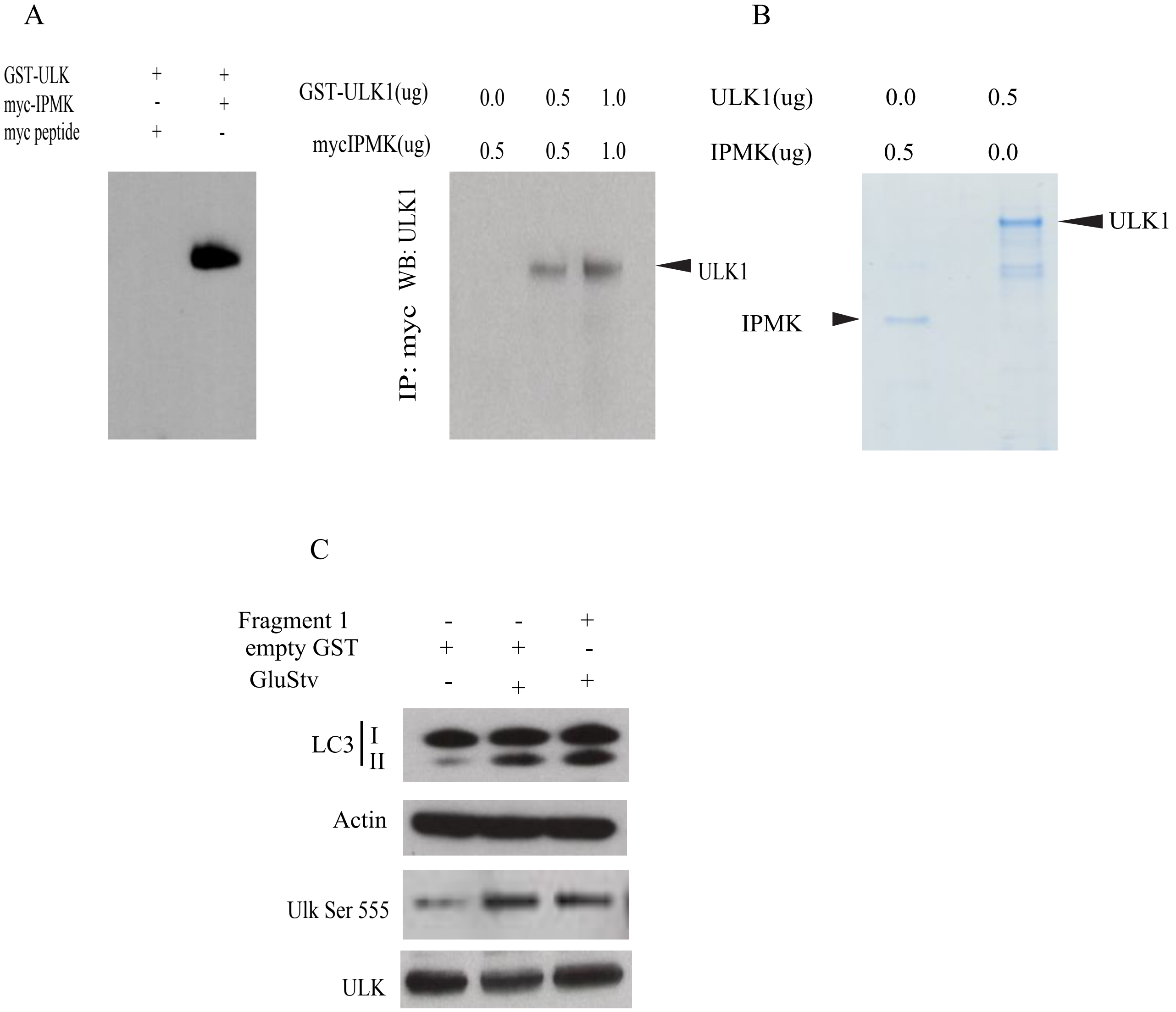

A
GST-ULK + +
myc-IPMK - +
myc peptide + -
GST-ULK1(ug) 0.0 0.5 1.0
mycIPMK(ug) 0.5 0.5 1.0
IP: myc WB: ULK1
ULK1
B
ULK1(ug) 0.0 0.5
IPMK(ug) 0.5 0.0
ULK1
IPMK
C
Fragment 1 - - +
empty GST + + -
GluStv - + +
LC3 I II
Actin
Ulk Ser 555
ULK

Figure S4. IPMK interacts with ULK1, Related to Figure 4.

(A) Recombinant mycIPMK was incubated with recombinant ULK1. Myc IPMK was immunoprecipitated and western blot performed for ULK1. (B) Validation of purity of recombinant protein in NuPAGE gel electrophoresis. (C) Fragment 1 of IPMK was transiently transfected in HEK293 cells followed by glucose starvation to analyze effects of fragment 1 on LC3II and ULK Ser 555.

Figure S 5

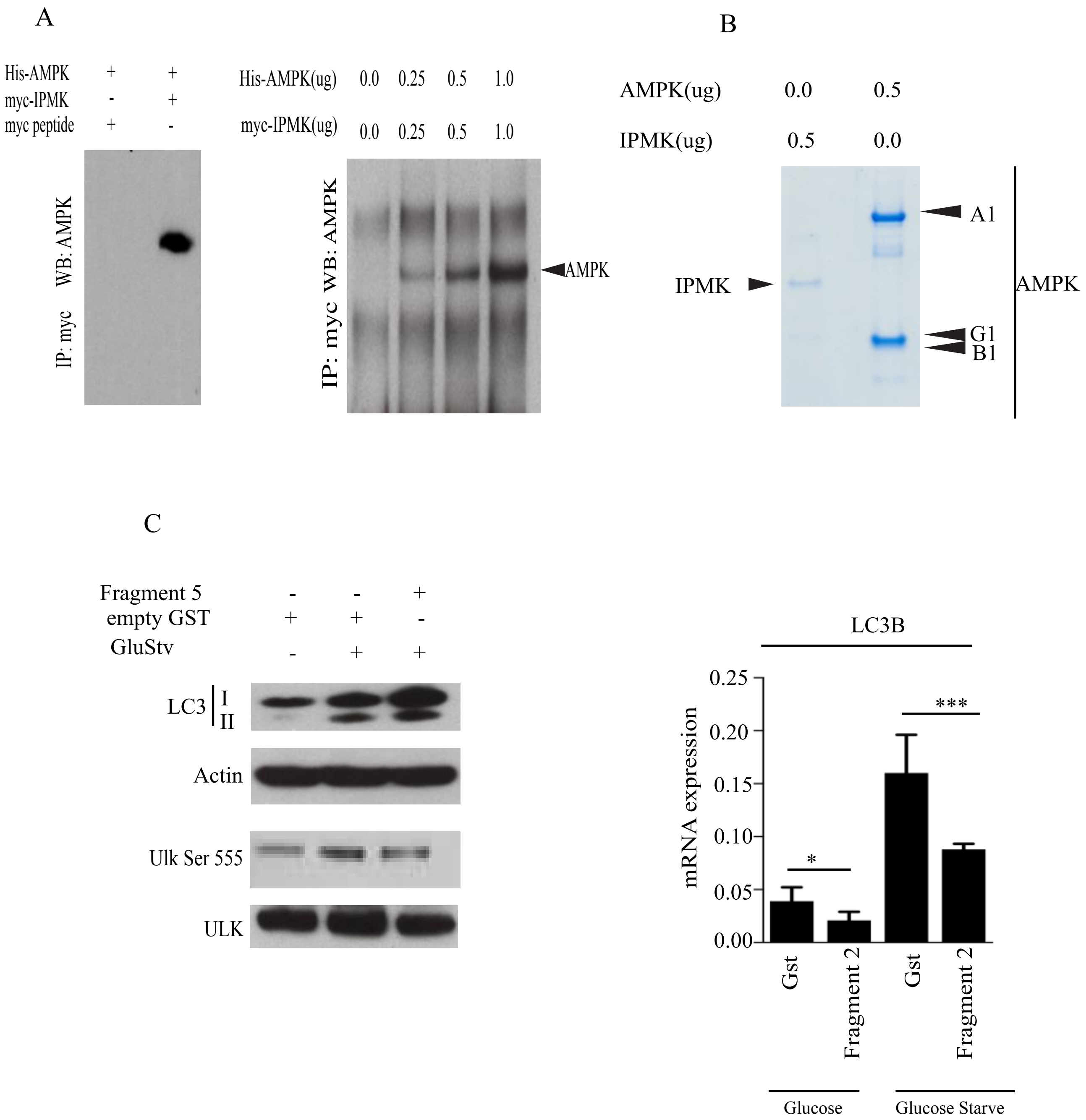

A
His-AMPK + +
myc-IPMK - +
myc peptide + -
IP: myc WB: AMPK
His-AMPK(ug) 0.0 0.25 0.5 1.0
myc-IPMK(ug) 0.0 0.25 0.5 1.0
IP: myc WB: AMPK
AMPK
B
AMPK(ug) 0.0 0.5
IPMK(ug) 0.5 0.0
IPMK
A1
G1
B1
AMPK
C
Fragment 5 - - +
empty GST + + -
GluStv - + +
LC3 I II
Actin
Ulk Ser 555
ULK
LC3B
mRNA expression
0.25
0.20
0.15
0.10
0.05
0.00
*
***
Gst
Fragment 2
Gst
Fragment 2
Glucose
Glucose Starve

Figure S5.

IPMK interacts with AMPK, Related to Figure 5.

(A) Recombinant mycIPMK was incubated with recombinant AMPK. Myc-IPMK was immunoprecipitated, and samples were western blotted for AMPK. (B) Validation of purity of recombinant protein in NuPAGE gel electrophoresis. (C) Fragment 5 of IPMK was transiently transfected in HEK293 cells followed by glucose starvation to analyze effects of fragment 1 on LC3II and ULK Ser 555. (D) Transcription of LC3B mRNA. $p<0.05$ *, $p<0.001$ *** . Data are means ± SD.

Figure S 6

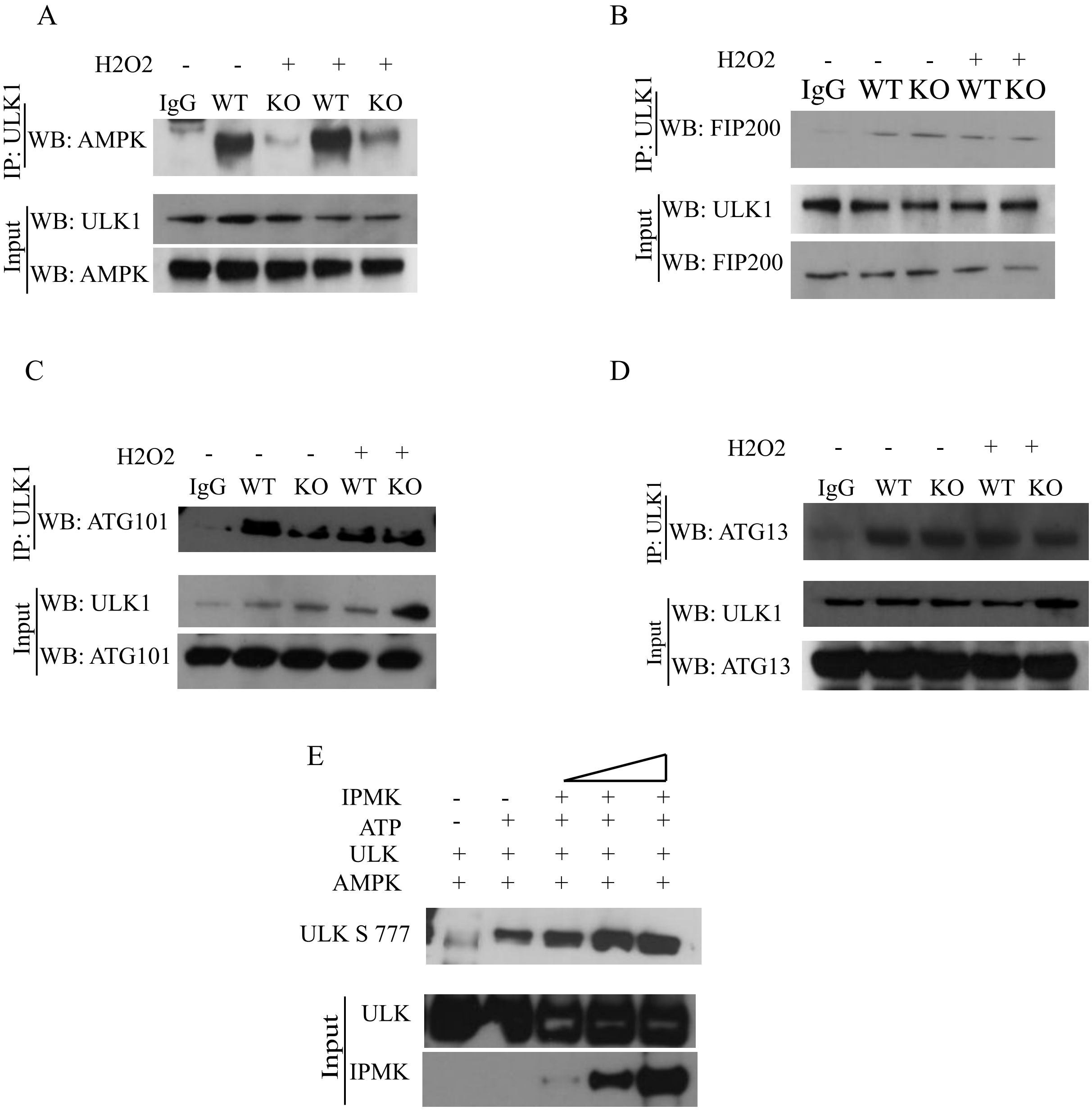

A
H2O2 - - + + +
IgG WT KO WT KO
IP: ULK1
WB: AMPK
Input
WB: ULK1
WB: AMPK
B
H2O2 - - - + +
IgG WT KO WT KO
IP: ULK1
WB: FIP200
Input
WB: ULK1
WB: FIP200
C
H2O2 - - - + +
IgG WT KO WT KO
IP: ULK1
WB: ATG101
Input
WB: ULK1
WB: ATG101
D
H2O2 - - - + +
IgG WT KO WT KO
IP: ULK1
WB: ATG13
Input
WB: ULK1
WB: ATG13
E
IPMK - - + + +
ATP - + + + +
ULK + + + + +
AMPK + + + + +
ULK S 777
Input
ULK
IPMK

Figure S6.

IPMK is essential for AMPK/ULK1 interactions, Related to Figure 6.

(A) IPMK wild type and KO MEFs were treated with H_2O_2 (500 μM). The role of IPMK as a scaffold was analyzed by immunoprecipitation of endogenous ULK1 and western blot of endogenous AMPK, (B) FIP200, (C) ATG101, (D) ATG13. In vitro AMPK dependent ULK phosphorylation, in presence of increasing amounts (100ng, 500ng, 1ug) of recombinant human IPMK (E).

Figure S 7

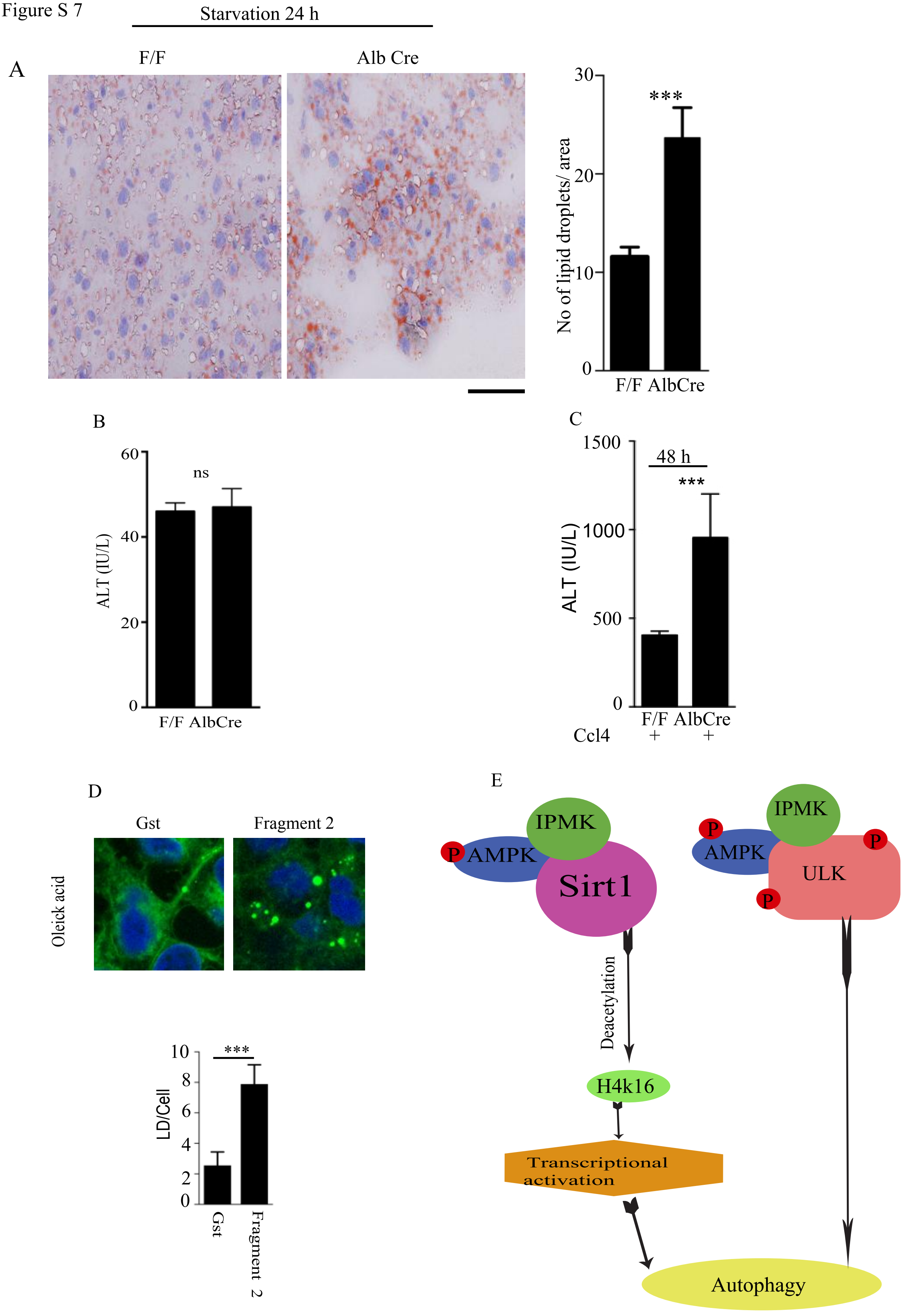

Starvation 24 h
A
F/F
Alb Cre
No of lipid droplets/ area
30
20
10
0
***
F/F AlbCre
B
ALT (IU/L)
60
40
20
0
ns
F/F AlbCre
C
ALT (IU/L)
1500
1000
500
0
48 h
***
F/F AlbCre
Ccl4
+
+
D
Gst
Fragment 2
Oleick acid
LD/Cell
10
8
6
4
2
0
***
Gst
Fragment 2
E
IPMK
P
AMPK
Sirt1
Deacetylation
H4k16
Transcriptional activation
IPMK
P
AMPK
P
ULK
P
Autophagy

Figure S7. IPMK is required for lipophagy and cytoprotection , Related to Figure 7.

(A)IPMK F/F and Alb (Cre) mice were starved for 24h and the livers harvested for Oild red O (ORO) staining to quantitate accumulated lipid droplets. Numbers of lipid droplets were counted and represented as bars. (n=3), p<0.001 ***. Data are means ± SD. Scale bar 200uM (B) Serum ALT level in untreated mice,(n=5), Data are means ± SD. (C)Serum ALT level in Ccl4 treated mice,(n=5), p<0.001 ***, Data are means ± SD. (D) Lipid droplet counts in HEK 293 cells after fragment 2 over expression. (E) Schematic diagram of IPMK mediated autophagy induction. IPMK interacts with target proteins to form signaling complexes. IPMK-AMPK-Sirt1 signaling axis enhances AMPK activation followed by Sirt1 dependent deacetylation of H4K16ac resulting in transcriptional activation of autophagy related genes. In the IPMK-AMPK-ULK signaling axis, IPMK forms a ternary complex and promotes AMPK dependent ULK phosphorylation leading to activation of autophagy.
